# A cellular senescence-related classifier based on a tumorigenesis- and immune infiltration-guided strategy can predict prognosis, immunotherapy response, and candidate drugs in hepatocellular carcinoma

**DOI:** 10.3389/fimmu.2022.974377

**Published:** 2022-11-15

**Authors:** Yi Luo, Hao Liu, Hong Fu, Guo-Shan Ding, Fei Teng

**Affiliations:** ^1^ Department of Liver Surgery and Organ Transplantation, Changzheng Hospital, Naval Medical University, Shanghai, China; ^2^ Department of General Surgery, Ruijin Hospital, Shanghai Jiao Tong University, School of Medicine, Shanghai, China

**Keywords:** cellular senescence, hepatocellular carcinoma, tumor microenvironment, prognosis, immunotherapy

## Abstract

**Background:**

Cellular senescence plays an irreplaceable role in tumorigenesis, progression, and tumor microenvironment (TME) remodeling. However, to date, there is limited research delineating the landscape of cellular senescence in hepatocellular carcinoma (HCC), and an improved understanding on the interaction of tumor-associated cellular senescence with HCC prognosis, TME, and response to immunotherapy is warrant.

**Methods:**

Tumorigenic and immune infiltration-associated senescence genes were determined by weighted gene co-expression network analysis (WGCNA) and the Estimation of STromal and Immune cells in MAlignant Tumor tissues using Expression data (ESTIMATE) algorithm, and subsequently, a prognostic scoring model (named TIS) was constructed using multiple survival analysis algorithms to classify the senescence-related subtypes of HCC. Gene set enrichment analysis (GSEA) and gene set variation analysis (GSVA) were conducted to identify the distinct hallmark pathways between high- and low-risk subtypes. Additionally, we carried out correlation analyses for TIS and clinical traits, senescence-associated secretory phenotype (SASP), immune infiltration and evasion, immune checkpoint factors, drug response, and immunotherapeutic efficacy. External experimental validation was conducted to delineate the association of CPEP3 (a TIS gene) with HCC phenotypes through assays of proliferation, colony formation, and invasion.

**Results:**

A five-gene TIS, composed of NET1, ATP6V0B, MMP1, GTDC1, and CPEB3, was constructed and validated using TCGA and ICGC datasets, respectively, and showed a highly robust and plausible signature for overall survival (OS) prediction of HCC in both training and validation cohorts. Patients in the TIS-high group were accompanied by worse OS, activation of carcinogenetic pathways, infiltration of immunosuppressive cells, exclusion of effector killing cells, overexpression of immunomodulatory genes and SASP, and unsatisfied response to immunotherapy. In response to anticancer drugs, patients in the TIS-high group exhibited enhanced susceptibility to several conventional chemotherapeutic agents (5-fluorouracil, docetaxel, doxorubicin, gemcitabine, and etoposide), as well as several inhibitors of pathways involved in cellular senescence (cell-cycle inhibitors, bromodomain and extraterminal domain family (BET) inhibitors, PI3K-AKT pathway inhibitors, and multikinase inhibitors). Additionally, four putative drugs (palbociclib, JAK3 inhibitor VI, floxuridine, and lestaurtinib) were identified as potential compounds for patients in the TIS-high group. Notably, *in vitro* functional validation showed that CPEB3 knockdown boosted the phenotypes of proliferation, clonogenicity, and invasion in HCC cells, whereas CPEB3 overexpression attenuated these phenotypes.

**Conclusions:**

Our study provides comprehensive clues demonstrating the role of novel TIS in predicting HCC prognosis, immunotherapeutic response, and candidate drugs. This work highlights the significance of tumorigenesis- and immune infiltration-related cellular senescence in cancer therapy.

## Introduction

Hepatocellular carcinoma (HCC) is a major contributor to the worldwide health burden with high morbidity and mortality. According to the most recent cancer statistics, 905,677 new HCC cases were diagnosed in 2020, and over half of the patients were older than 60 years (1, 2). Despite the increasingly expanded indications for surgical and locoregional therapies, an estimate of 50%–60% HCC patients ultimately required systemic treatments (3). Currently, immunotherapy has emerged as the mainstay therapeutic paradigm for advanced-stage HCC whose efficacy is largely determined by the tumor microenvironment (TME), and regrettably, only a small proportion of patients presented clinical benefit (3). Therefore, it is imperative to identify novel classifiers or therapeutic biomarkers to delineate the immuno-oncology landscape and predict the benefit stratification of immunotherapy.

Cellular senescence refers to a physiological status of cell cycle arrest in response to endogenous and exogenous stress, characterized by persistently ceased proliferation but retained metabolic activity (4). Accumulative evidence has indicated that cellular senescence governs a vital role in aged-associated chronic liver diseases and even cancer through inducing a senescence-associated secretory phenotype (SASP) (5–7). Senescent cells can perform predominant SASP-mediated double-edged effects on neighboring cells and microenvironment remodeling to play both pro-tumorigenic and antitumorigenic functions, mainly depending on the physiological context of the microenvironment (8). At the early tumorigenic stages, cellular senescence functions as a tumor suppressor *via* immune activation and TME remodeling. However, when senescent cells are not eliminated by activated immune cells and accumulate at advanced phases, the special SASP of maladaptive senescence would enhance tumorigenic properties through epithelial-to-mesenchymal transition (EMT), angiogenesis, and extracellular matrix degrading signal, which activate immunosuppression, boost cell proliferation, drive tumor vascularization, and favor tumor progression (4, 9). In addition to tumorigenic senescent neighboring cells, tumor cells also acquire senescence as a malignant phenotype in response to the temporal cascade in the accumulation of SASP (9, 10). Consequently, cellular senescence is now under investigation as a therapeutic target of interest as specified *via* elimination of accumulated detrimental senescence and induction of acute cellular senescence. However, to date, there is limited research delineating the landscape of cellular senescence in HCC, and an improved understanding of the interaction of tumor-associated cellular-senescence with TME, prognosis, and response to immunotherapy is required in the HCC setting.

In the present study, we firstly identified tumor-associated cellular senescence genes based on weighted gene co-expression network analysis (WGCNA). Subsequently, we further filtered immune-associated senescent genes and established an innovative risk model for prognostic prediction of HCC. On the basis of the risk model, we next focused on the analysis of the landscapes of risk subgroups with tumor stages, immune infiltration and evasion, immunotherapy response, and potential therapeutic drugs.

Finally, we conducted experimental verification to delineate the senescence-oncology role of CPEB3 (one of the model genes). Our study provided novel insights into the substantial immune-oncology properties of cellular senescence in TME remodeling and for immunotherapy prediction for HCC patients.

## Materials and methods

### Data acquisition and processing

Due to no definite composition about tumor-associated senescence genes in light of the current studies, an expanded exploration was determined to identify tumor-associated senescence genes based on high-quality databases and published literatures, including integrating subsets from HAGR (https://genomics.senescence.info/genes/index.html) (11), SenMayo gene set (https://www.biorxiv.org/content/10.1101/2021.12.10.472095v1), and MSigDB (http://www.gsea-msigdb.org/gsea/msigdb/index.jsp) (12).

Transcriptomic and clinical data of HCC patients were extracted from The Cancer Genome Atlas (TCGA, http://cancergenome.nih.gov) and International Cancer Genome Consortium (ICGC, https://dcc.icgc.org/releases/current/Projects/LIRI-JP). For TCGA dataset, normalized transcripts with log-transformed transcripts per million (TPM) were employed for downstream analyses, and a total of 343 HCC patients with censored survival time >30 days were enrolled as a training cohort for model establishment (13). Similarly, a total of 231 patients with complete survival time and status from the ICGC-LIRI-JP dataset were employed as the validation cohort, in which transcriptomic data were normalized in the form of log2(TPM+1).

**Figure 1:**
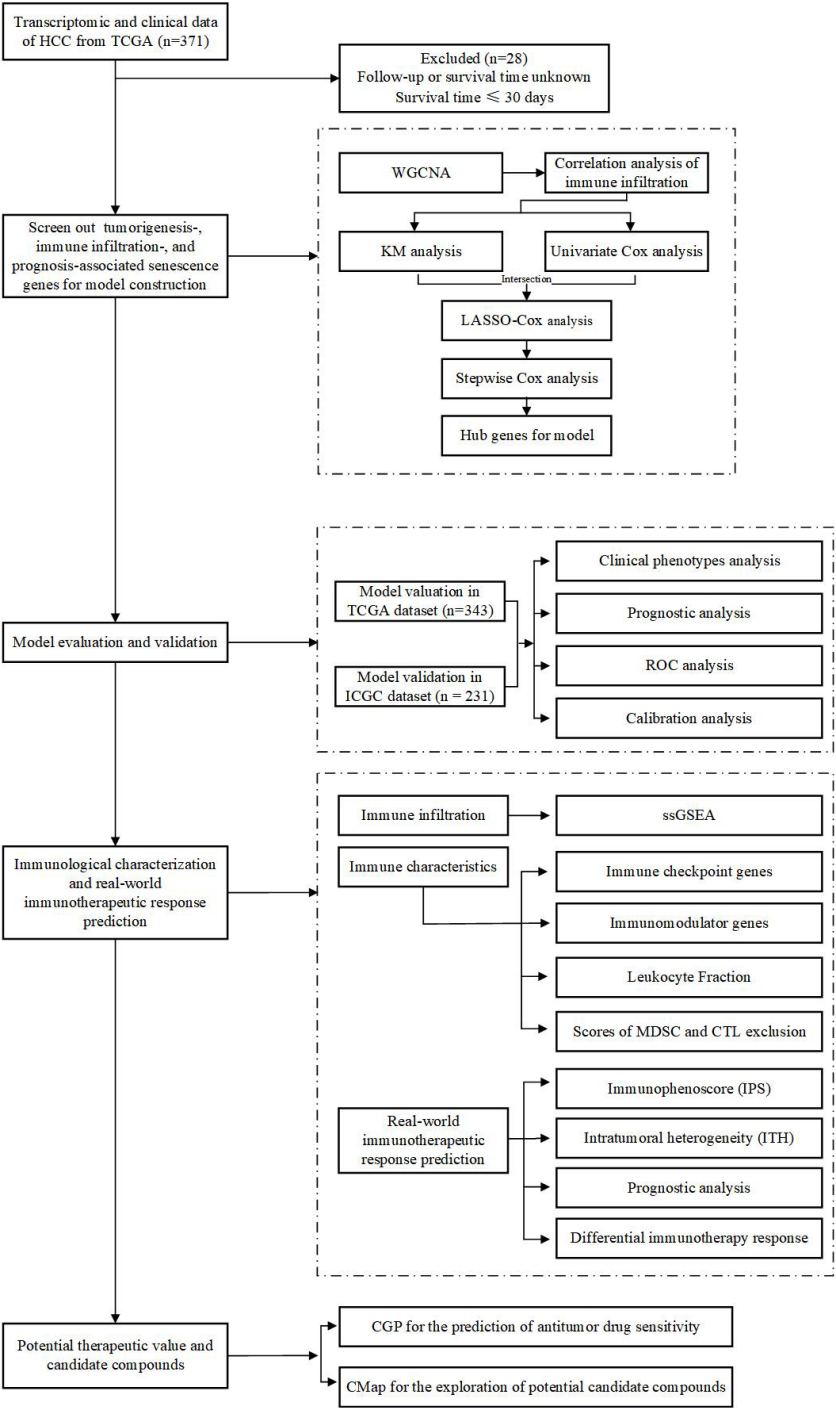
Flowchart of the overall study design.

An immune checkpoint blockade (ICB) dataset containing anti-CTLA4, anti-PD1, and anti-PDL1 cohorts was derived from Cancer Research Institute (CRI) iAtlas (https://isb-cgc.shinyapps.io/iatlas/), in which log2(normalized count+1) was used for data normalization. Patients in this study were derived from 12 integrated independent cohorts across skin cutaneous melanoma (SKCM), kidney renal clear cell carcinoma (KIRC), bladder urothelial carcinoma (BLCA), stomach adenocarcinoma (STAD), glioblastoma multiforme (GBM), and head and neck squamous cell carcinoma (HNSC), and a total of 871 patients with complete survival time and status were included in our study (14–25). The flow diagram of this study is depicted in **Figure 1**.

### Identification of tumorigenic and immune infiltration-associated senescence genes by WGCNA and ESTIMATE algorithms

Following gene filtering with expression values >0 in at least one quarter of TCGA samples, a total of 1,889 senescence genes (**Supplementary Table S1**) were enrolled to perform WGCNA. R package “WGCNA” was employed to construct the weighted adjacency matrix and the topological overlap matrix (TOM) to determine the correlation among senescence genes. Stringent criteria were adopted to excavate the interconnected modules with a minimal module size of 30, split depth of 3, and merged threshold of 0.25. The module–phenotype relationship was calculated to identify the gene sets most relevant to the tumor phenotype, and selected senescence genes were extracted for subsequent analysis.

It was documented that the tumor-immune microenvironment could directly impact cellular senescence’s pro-tumorigenic or antitumorigenic tendency, as well as therapy response (26). Therefore, immune infiltration-related cellular senescence genes were emphatically concerned in this study. Estimation of STromal and Immune cells in MAlignant Tumor tissues using Expression data (ESTIMATE) was utilized to evaluated the immune cell infiltration level quantified by immune score. The correlation between immune score and tumorigenic senescence genes was calculated, and genes were considered when *P* < 0.001.

### Construction and validation of the tumorigenic and immune infiltration-associated senescence signature

The aforementioned senescent genes were computed with univariate Cox regression and Kaplan–Meier (KM) analyses with overall survival (OS) as the censored endpoints, employing R package “survival” and “survminer”. Subsequently, the intersections of prognostic genes were calculated by Least Absolute Shrinkage and Selection Operator (LASSO) Cox regression analysis using package “glmnet”, and lambda.min was determined to pick up the preliminary hub genes following 10-fold cross validation and a 1,000-times repeat (27). Final hub senescence genes of tumorigenic and immune infiltration-associated senescence signature (TIS) were subjected to forward stepwise Cox regression to further narrow and simplify variables. An individual risk score was generated on the basis of hub gene expression and corresponding regression coefficients. In light of the median risk score computed by TIS, patients in the training and validation cohorts were dichotomized into high- and low-risk groups. A visual differential expression of the five signature genes between tumor and para-cancerous tissues as well as between high- and low-risk groups was modeled utilizing TCGA RNA-sequencing dataset. Moreover, corresponding KM curves were drawn to delineate the prognostic landscape of signature genes, with OS parameter as the censored endpoint. The performance of TIS was subsequently explicitly evaluated by receiver operating characteristic (ROC) and calibration analyses in both training and validation cohorts, using R packages “timeROC” and “rms”, respectively. Additionally, the differential distribution of clinical characteristics, covering age, gender, clinicopathological grade, TNM stage, and survival status, between the TIS-high and TIS-low groups was separately deciphered and visualized in TCGA and ICGC datasets.

### Construction of a TIS-integrated nomogram

Univariate and multivariate Cox analyses were utilized to identify the independent predictive and prognostic potential of TIS in HCC, and forest plots were employed for visualization with R package “forestplot”. Subsequently, a quantitative TIS and TNM stage integrated nomogram was generated to compute individualized risk for HCC patients. Calibration plot, the area under the ROC curve (AUC), and decision curve analysis (DCA) were utilized to determine the TIS performance with “rms”, “timeROC”, and “ggDCA” packages, respectively. Furthermore, the KM diagram of the TIS-integrated nomogram was delineated in TCGA dataset, when OS, disease-special survival (DSS), progression-free interval (PFI), and disease-free interval (DFI) were employed as the censored endpoints.

### Pathway enrichment analysis with gene set enrichment analysis and gene set variation analysis

In the current study, gene set enrichment analysis (GSEA) was implemented to decipher the underlying mechanism of TIS with regard to the 50 hallmark pathways (v7.5.1) deposited in the molecular signature database, whose result was computed with the “clusterProfiler” package and was visualized with the “enrichplot” package (28, 29).

The gene set variation analysis (GSVA) enrichment score of the above 50 oncogenic pathways for each patient was determined using the “GSVA” package (30). Subsequently, KM diagrams were operated to determine the prognostic pattern of the top overlapping oncogenic pathways of GSVA and GSEA.

### The association of TIS with immune infiltration and immunomodulatory genes

Single-sample gene set enrichment analysis (ssGSEA) could determine the relative infiltration of 28 immune cell types and two stromal components (fibroblasts and endothelial cells) based on immune deconvolution analyses with special feature gene panels for each immune and stromal cell subset, and the relative abundance of each cell type of each tumor sample was represented by an enrichment score which can be used for subsequent analyses (31). In the present research study, we introduced ssGSEA to quantify the abundance of 28 immune cells for each TCGA sample between TIS-high and TIS-low groups (32). Beyond this, key immune characteristics (33), including leukocyte fraction, loss of heterozygosity (LOH), homologous recombination deficiency (HRD), and intratumor heterogeneity (ITH), were downloaded from https://gdc.cancer.gov/about-data/publications/panimmune and were compared between the TIS-high and TIS-low groups. The scores of myeloid-derived suppressor cell (MDSC) and cytotoxic T lymphocyte (CTL) exclusion were downloaded from Tumor Immune Dysfunction and Exclusion (TIDE, http://tide.dfci.harvard.edu/) (34). Heatmap, chordal graph, and boxplots were implemented to delineate the correlation of TIS with immune checkpoint genes and 50 immunomodulatory genes.

### Prediction of immunotherapeutic response

Immunophenoscore (IPS), which has been documented with favorable predictive power to ICB response, was downloaded from The Cancer Immunome Atlas (https://tcia.at), with a higher score referring to a higher response to ICB (32). Subsequently, the differential distribution between the TIS-high and TIS-low groups was visualized with violin plots. To verify the response-efficacy prediction of TIS in real-world data, an immunotherapeutic dataset of solid tumors was also dichotomized into the TIS-high and TIS-low groups according to the corresponding regression coefficients of the TIS model. Four response-efficacy indices of immunotherapy, namely, progressive disease (PD), stable disease (SD), partial response (PR), and complete response (CR), were employed to evaluate the predictive power. A KM plot was described to decipher the prognostic pattern of the TIS-high and TIS-low group in the real-world cohort. Box and bar plots were performed to identify the differential distribution of risk score and response-efficacy indices between the TIS-high and TIS-low groups.

### Prediction of antitumor drug sensitivity and potential candidate compounds

Drug response information, measured with AUC across various cancer cells, of 212 drugs for HCC was obtained from the Cancer Genome Project (CGP) *via* R package “pRRophetic” (35). Normalized half-maximal inhibitory concentrations (IC50) of each TCGA sample were quantified. A Boxplot was carried out to capture the differential drug sensitivity between the TIS-high and TIS-low groups.

In order to figure out the putative drug for TIS-high patients, we performed chemotherapeutics forecast *via* the “query” module of the connectivity map (CMap, https://clue.io/query) (36). Following uploading of the upregulated and downregulated genes between the TIS-high and TIS-low groups, permuted results were obtained, and subsequently, the 2D and 3D drug structures of the top four potential compounds were further visualized *via* the PubChem website (https://pubchem.ncbi.nlm.nih.gov/).

### Cell culture and lentivirus transfection

Human-derived hepatoma cells (Hep3B, SNU-182, SNU-387, Huh-7, SKHEP1), human embryonic kidney-293T cells (HEK-293T), and human immortalized hepatocytes (L02) were obtained from the National Key Laboratory of Medical Immunology and Institute of Immunology, Navy Medical University. Except SNU-182 and SNU-387 which were cultured in Roswell Park Memorial Institute 1640 (RPMI-1640, Gibco, 11875093), all cells were maintained in Dulbecco’s modified Eagle’s medium (DMEM, Gibco, 11095092) supplemented with 10% fetal bovine serum (Gibco, 10099141), 100 IU/ml penicillin, and 100 µg/ml streptomycin.

Lentivirus carrying full-length CPEB3 mRNA (NM014912) followed by 1 × 3′-FLAG tag, short hairpin RNA (shRNA) sequences against CPEB3, and corresponding negative control (NC) sequences were constructed by Tsingke Biological Technology (Nanjing, China) and are available in **Supplementary Table S2**. For stable cell line establishment, a Hep3B cell was transfected with the indicated lentivirus with 6 µg/ml polybrene, and 72 h after transfection, a final concentration of 4 ug/ml puromycin was added to screen the positive cells for 7 days.

### RNA extraction, cDNA synthesis, and qRT-PCR

Total RNA was extracted using an RNA isolation kit (Vazyme Biotech, RC112-01) and was subsequently reverse transcribed into cDNA using the PrimeScript RT reagent Kit (TAKARA, RR036A) according to the manufacturer’s instructions. All qRT-PCR reactions were performed in triplicate with β-actin expression as a normalized internal reference, using SYBR Premix Ex Taq (TAKARA, RR420A). The PCR primers are listed in **Supplementary Table S3**.

### Western blotting

Detailed procedures for Western blot were performed as previously described (37). The characteristics and working dilutions of the antibodies used are provided in **Supplementary Table S4**.

### Cell proliferation assay

Hep3B cells and stably transfected Hep3B cells were seeded into 96-well plates at a concentration of 4,000 cells/well. Cell proliferation capacity was assessed at 24, 48, 72, and 96 h by CCK-8 assays (Topscience, C0005).

### Colony formation assay

To determine the capacity of population dependence and clonal proliferation, cells were seeded in a six-well plate at a density of 5.0 × 10^2^/well and continuously incubated for 14 days. Colony fixation was done with methanol for 20 min, and subsequently, colonies were stained with 0.2% crystal violet for 30 min. ImageJ software was employed for colony count.

### Matrigel invasion assay

A Transwell chamber (8-μm pore size, Corning Incorporated, 3422) covered with 40 µl BD Matrigel (diluted 1:8 with serum-free medium) was used for cell invasion assay. Hep3B cells (6.0 × 10^4^) suspended in 200 μl of FBS-free medium were seeded in the upper chamber, and 600 µl medium containing 10% FBS was added to the lower chamber. Following a 36-h incubation, cells adhering to the lower filter surface were fixed, stained, and counted. Of note, cells were pretreated with 10 µg/ml of mitomycin c (Selleck Chemicals, S8146) for 2 h to eliminate the effect of cell proliferation.

### Statistical analysis

R software (version 4.1.3) was implemented for public data processing, statistical analysis, and diagram formation. Differential distributions between two groups were determined by the Wilcoxon’s test. Differential survival probability was visualized utilizing KM analysis and log-rank test. Hazard ratios (HRs) were employed in univariate and multiple Cox regression analyses. Pearson’s correlation test and Student t test were employed in correlation analysis of the module gene significance, clinical traits, immune infiltration and evasion, and expression of immune checkpoint fators. All experiments were performed in triplicates and expressed as mean ± SEM using GraphPad Prism (8.0.2). Statistical significance was determined by t test (two-tailed) for two groups or one-way ANOVA for three or more groups. All results were deemed as statistically significant with two-sided *P* < 0.05.

## Results

### Identification of tumorigenic and immune infiltration-related senescence genes

**Figure 2:**
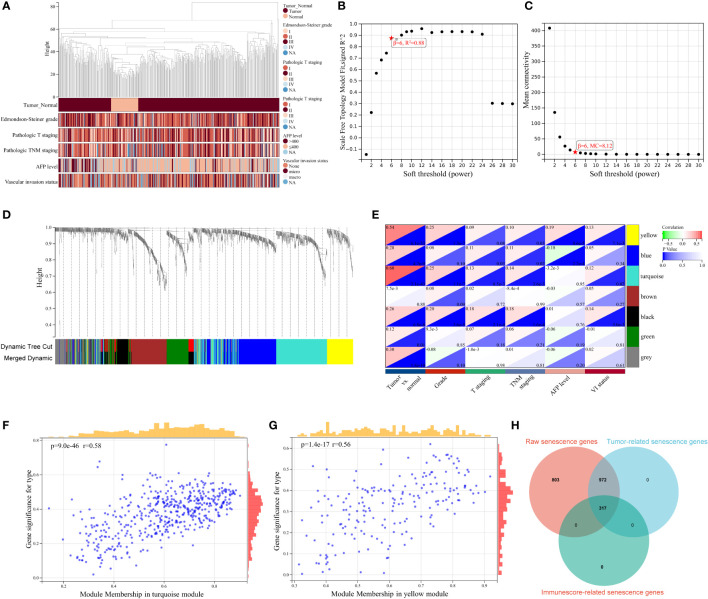
WGCNA for identification of tumor-related senescence genes. **(A)** Clustering dendrogram of HCC samples and the clinical traits, covering sample type (tumor and normal), Edmondson–Steiner grade, pathologic T staging, pathologic TNM staging, alpha fetoprotein (AFP) level, and vascular invasion (VI) status. Soft threshold selection to determine the WGCNA module depending on scale independence **(B)** and mean connectivity **(C)**. **(D)** Seven colored modules were determined and visualized with a dendrogram, based on a dissimilarity measure (1-TOM). **(E)** Heatmap for the correlation between gene modules and clinical characteristics. Scatter plots delineating the correlation between gene significance (GS) for tumor trait and module membership (MM) in the turquoise **(F)** and yellow module **(G)**. **(H)** Venn diagram showing 317 overlapping tumorigenesis- and immune infiltration-associated genes. WGCNA, weighted gene co-expression network analysis; MC, mean connectivity; NA, not available.

Given that cellular senescence governs key aspects of chronic diseases and cancer, in our HCC-associated study, we focused specially on tumor-relevant senescence genes. WCGNA was first harnessed for sample clustering to identify outlier samples, and no sample was determined for removal from the study (**Figure 2A**). A scale-free network was constructed with soft thresholding power set as 6 (scale-free R^2^ = 0.88), in which scale independence and mean connectivity presented a relative balance (**Figures 2B, C**). As shown in the cluster dendrogram, a total of seven modules (i.e., turquoise, yellow, blue, black, brown, green, gray) were detected following similar module integration (**Figure 2D**). Notably, the gray module represents taxonomic genes deserting from the other module genes. On the basis of the correlation results between clinical characteristic and module, statistically significant positive correlations of module with tumor and pathological stage characteristics were distinguished in module turquoise, yellow, blue, and black (all *P* < 0.05, **Figure 2E**), and scatter plots were performed to delineate the correlation between tumor trait and turquoise genes (**Figure 2F**, r > 0.5, *P* < 0.001), as well as yellow genes (**Figure 2G**, r > 0.5, *P* < 0.001). Previous studies have denoted the pervasive regulatory pattern of cellular senescence on immune cell infiltration across multiple cancers (4, 38, 39). Consequently, we introduced the “ESTIMATE” algorithm to quantify the immune cell infiltration landscape for all tumor samples, and 317 immune infiltration-related senescence genes (**Supplementary Table S5**) were ultimately determined through calculating the correlation between immune score and 1,289 WGCNA genes (**Supplementary Table S6**) with a *P* value lower than 0.001 (**Figure 2H**). In this section, tumorigenic and immune infiltration-related senescence genes were addressed and described, and filtered senescence genes were preliminarily considered for downstream signature identification.

### Construction of TIS and signature gene analyses

**Figure 3:**
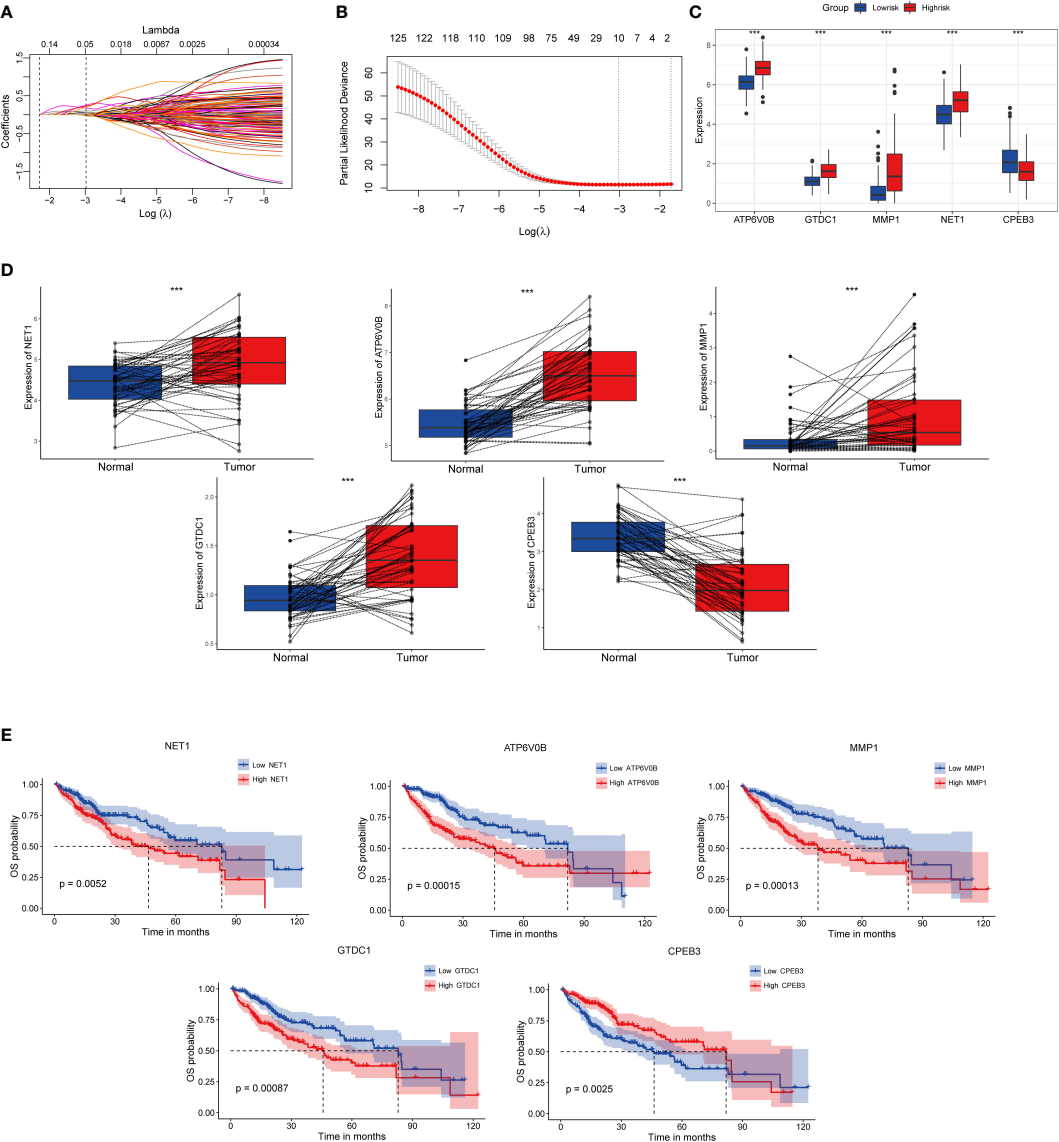
Identification, expressed pattern, and survival analysis of TIS genes. **(A)** LASSO coefficient profiles of 127 genes. **(B)** Cross-validation for tuning parameter selection in the LASSO regression. Expressed divergence of TIS genes between high-risk and low-risk groups **(C)** as well as between cancer and corresponding para-cancerous tissues **(D)**. **(E)** KM plots of OS were performed to elaborate the prognostic value of TIS genes. KM plots, Kaplan–Meier plots; OS, overall survival; *P < 0.05; **P < 0.01; ***P < 0.001.

In order to construct a high-performance senescence-related prognostic signature, we first entered all the above 317 genes for both KM and univariate Cox regression analyses. Subsequently, 127 intersection-prognostic genes (**Supplementary Table S7**) were inputted into LASSO–Cox regression analysis for stringent feature selection, and 10 genes selected at lambda.min were further streamlined and optimized through forward stepwise Cox regression (**Figures 3A, B**). A final five robust genes were incorporated into TIS construction for predicted HCC prognosis. The TIS risk score formula was determined as the following: risk score = (0.5019268 * expression value of NET1) + (0.5922202 * expression value of ATP6V0B) + (0.1514856 * expression value of MMP1) + (0.4845483 * expression value of GTDC1) + (-0.2477429 * expression value of CPEB3). HCC samples from the training and validation cohorts were dichotomized into TIS-high and TIS-low groups according to the median value of risk score in the two datasets, respectively.

Compared with the low-risk group, NET1, ATP6V0B, MMP1, and GTDC1 showed elevated expression abundance in the high-risk group, whereas CPEB3 exhibited the opposite expression profile (**Figure 3C**, *P* < 0.001). Additionally, consistent expression tendencies were observed in paired tumor and para-cancerous tissues, which indicated that signature genes might be involved in HCC progression and metastasis (**Figure 3D**). Afterward, we investigated the influence of individual signature genes on OS possibility and found that elevated expressions of NET1, ATP6V0B, MMP1 and GTDC1 substantially contributed to worse OS, whereas CPEB3, contrary to the above genes, might play a protective role in HCC setting (**Figure 3E**).

### Favorable performance of TIS for OS prediction

**Figure 4:**
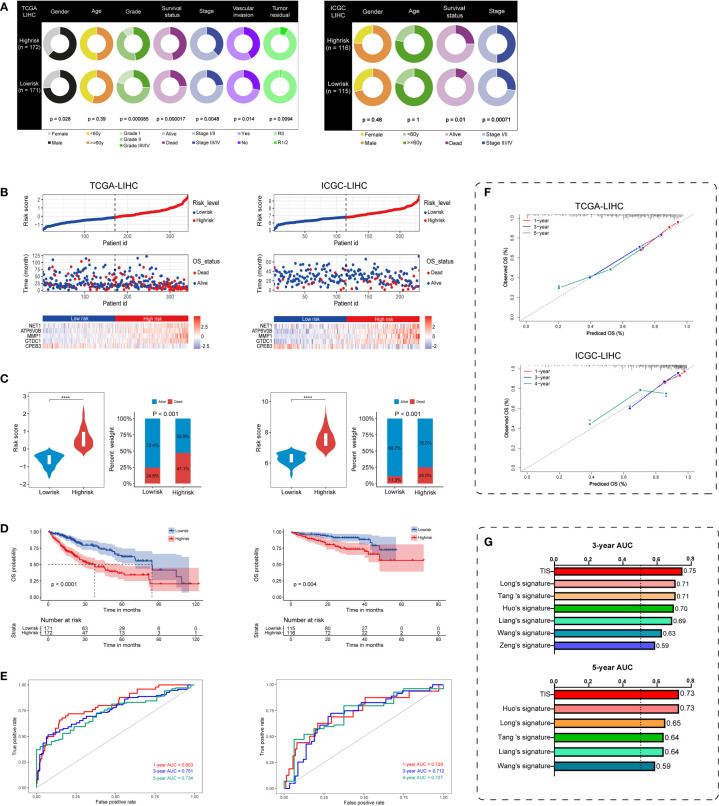
Performance evaluation and validation of the TIS in HCC. **(A)** Differential correlation distribution of clinical traits between high- and low-risk groups in TCGA database (left) and ICGC database (right). **(B)** Distribution of risk score, survival status, and expressed profiles of TIS genes in low- and high-risk groups from TCGA training dataset and ICGC validation dataset. **(C)** Risk score and mortality rate of patients in high- and low-risk groups in two datasets. **(D)** KM plots of OS showing the discriminated survival between high- and low-risk groups in two datasets. ROC curves with AUC values **(E)** and calibration plots **(F)** were employed to elaborate TIS performance in two datasets. **(G)** Distinct 3- and 5-year AUC of TIS and several published signatures. TCGA, The Cancer Genome Atlas; ICGC, International Cancer Genome Consortium; ROC, receiver operating characteristic; AUC, the area under the ROC curve; ****P < 0.0001.

On the basis of TIS, we proceeded with multi-index annotation to evaluate and validate model performance in TCGA and ICGC datasets. As illustrated in **Figure 4A**, survival outcomes and pathologic traits exhibited a differential distribution in the high- and low-risk groups in both datasets, and high-risk patients presented increased exposure to worse pathologic stage and death (*P* < 0.01). Furthermore, plots for the distribution of TIS risk score, survival status, and TIS gene expression profiles in low- and high-risk groups were delineated to capture the influential pattern of TIS (**Figure 4B**). These results indicated that a higher risk score contributes to a higher mortality and a higher expression level of carcinogenic TIS genes. Patients in the high-risk group had nearly twice the mortality in the low-risk group (**Figure 4C**). Survival analysis revealed that patients in the low-risk group had a significantly better OS than those in the high-risk group in both training and validation cohorts (**Figure 4D**). Afterward, ROC analysis was employed to assess TIS performance, with AUC values of 0.803, 0.751, and 0.734 at the 1-, 3-, and 5-year censored endpoints in TCGA dataset and 0.729, 0.712, and 0.727 at the 1-, 3-, and 4-year censored endpoints in the ICGC dataset, respectively (**Figure 4E**). Additionally, a calibration plot also exhibited favorable predictive performance at different censored endpoints (**Figure 4F**). Of note, compared with several published models (40–45), TIS containing only five signature genes showed non-inferior performance with 3- and 5-year AUC values (**Figure 4G**). These results demonstrated that TIS was a highly robust and plausible signature for OS prediction of HCC, and an elevated TIS score was correlated with malignant phenotypes of tumorigenesis and progression in HCC.

### Independent prognostic value of TIS and construction of a TIS-based nomogram

**Figure 5:**
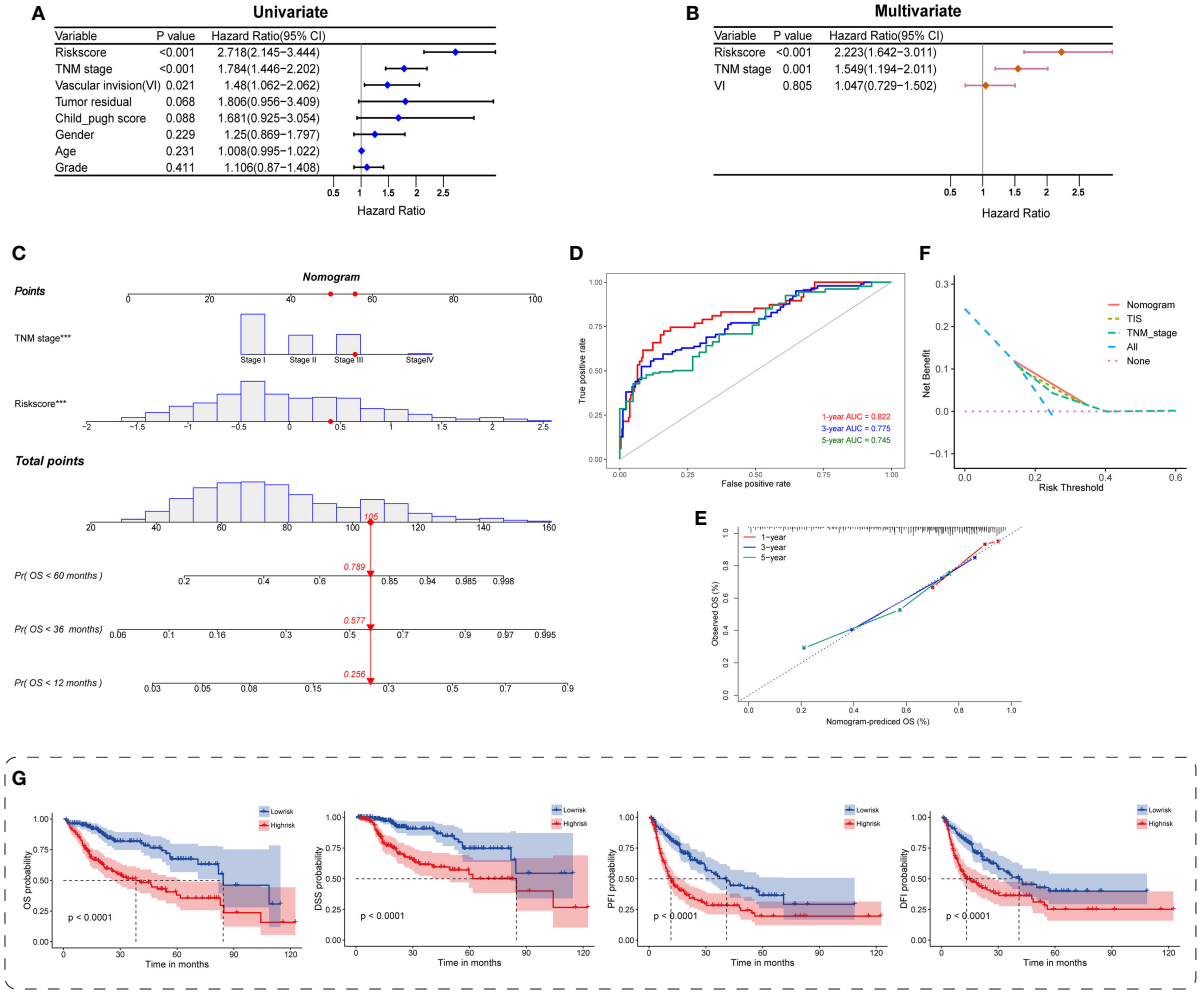
Construction and evaluation of the TIS-integrated nomogram. Forest plots for univariate **(A)** and multivariate Cox analyses **(B)** of TIS and clinical characteristics. **(C)** Nomogram incorporating TIS and pathological stage for predicting the 1-, 3-, and 5-year mortality in HCC. ROC curve **(D)** and calibration plot **(E)** for predicting the 1-, 3-, and 5-year performance of the nomogram. **(F)** Distinct net benefits of decision curves among nomogram, TIS, and TNM stage. **(G)** KM survival plots of the integrated nomogram for OS, DSS, PFI, and DFI. DSS, disease-specific survival; PFI, progression-free interval; DFI, disease-free interval.

To figure out the independent predictive potential of TIS for prognosis in HCC, we carried out univariate and multivariate analyses with multiple clinical traits covering age, gender, tumor pathological grading and staging, vascular invasion, residual tumor status, and Child–Pugh stage. The TIS score, TNM stage, and vascular invasion were preliminarily identified to be associated with OS (*P* < 0.05), and these indicators were subsequently enrolled in multivariate analysis (**Figure 5A**). Both TIS score and TNM stage functioned as independent and robust prognostic markers, and the TIS score exhibited more potency than the TNM stage (**Figure 5B**; HR = 2.223 and 1.549, respectively).

Next, we provided a quantitative TIS-integrated nomogram to compute individualized risk for HCC patients (**Figure 5C**). Three goodness-of-fit indices, namely, ROC, calibration, and DCA, were applied for model evaluation. The 1-, 3-, and 5-year AUCs for the TIS-integrated nomogram were 0.822, 0.775, and 0.745, respectively (**Figure 5D**). A calibration plot also presented ideal consistent prediction at the 1-, 3-, and 5-year censored OS (**Figure 5E**). Compared with TIS or TNM stage alone, the nomogram yielded a better net benefit, indicating the synergistic predictive power of TIS with TNM stage (**Figure 5F**). Beyond the above delineations, we also discovered the strong prognostic value of the TIS-integrated nomogram in HCC, when OS, DSS, PFI, and DFI were employed as the censored endpoints separately (**Figure 5G**). Hence, consistent with the previous description, TIS could serve as a core candidate predictor for HCC phenotypes and survival.

### Potential carcinogenetic mechanisms and SASP landscapes

**Figure 6:**
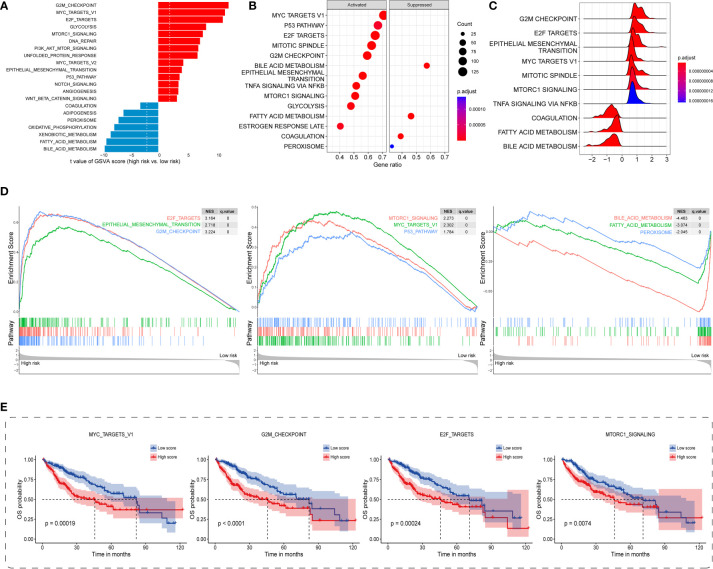
Distinct carcinogenetic mechanisms between TIS-high and TIS-low groups. **(A)** Bar graph for the differential enrichment in carcinogenetic pathways, determined by GSVA, between the TIS-high and TIS-low groups. Bubble plot **(B)** and ridge plot **(C)** for the differential enrichment in carcinogenetic pathways, determined by GSEA, between the TIS-high and TIS-low groups. **(D)** GSEA enrichment plots showing significantly enriched pathways including six upregulated and three downregulated pathways. **(E)** KM plots of OS delineating the prognostic landscapes of the four typical oncogenic pathways. GSVA, gene set variation analysis; GSEA, gene set enrichment analysis.

Regarding the underlying downstream mechanism between the TIS-high and TIS-low subgroups, we performed GSVA and GSEA to identify differential cancer hallmark pathways using TCGA transcriptomic dataset. On the basis of GSVA results (**Supplementary Table S8**), a total of 42 significantly differential pathways were sorted out utilizing the limma algorithm, of which 32 were upregulated and 10 were downregulated in the high-risk group. As 21 top enriched pathways shown in **Figure 6A**, the high-risk group is predominantly accompanied by the activation of carcinogenetic pathways compared with the low-risk group, such as G2M checkpoint, MYC, E2F, mTORC1, EMT, and PI3K-AKT-mTOR. Interestingly, the high-risk group presented evident metabolic dysregulation with upregulated glycolysis and downregulated oxidative phosphorylation, fatty acid metabolism, and adipogenesis (**Figure 6A**). GSEA also identified 39 significantly altered cancer hallmark pathways, of which 34 were upregulated and five were downregulated (**Supplementary Table S9**). Consistent with the GSVA results, the high-risk group exhibited primarily enhanced carcinogenetic pathways and attenuated non-glycolysis metabolic pathways (**Figures 6B-D**). In order to discover the prognostic landscape of upregulated hallmark pathways, we delineated survival plots for several common significant pathways including MYC, G2M checkpoint, E2F, and mTORC1. As expected, HCC patients with these activated pathways were characterized by a worse prognosis (**Figure 6E**). Taken together, the TIS-high subtype exhibited evident activation of multiple oncogenic pathways involved in tumorigenesis and metastasis, which resulted in unsatisfied prognosis.

In consideration of the irreplaceable role of SASP in tumor recurrence and progression, and TME remodeling (46–49), we also delineated the altered landscape of SASP, proteins secreted by senescent cells, between TIS-high and TIS-low groups. Noticeably, elevated SASP was found to be highly enriched in the TIS-high group (**Figures S1A-E**), including interleukins (IL-1A, IL-1B, IL-15, and IL7), soluble or shed receptors or ligands (PLAUR, ICAM1, TNFRSF11B, and TNFRSF10C), proteases and regulators (MMP14, MMP1, MMP12, and MMP10), chemokines (CXCL3, CXCL8, CXCL5, and VEGF), and growth factors and regulators (IGFBP3, PIGF, ANG, and EREG). Of these upregulated SASP, some, such as CXCL8 and VEGF, were documented to possess immunosuppressive properties (50). Therefore, we speculated that patients in the TIS-high group might be accompanied by detrimental oversecreted SASP and thereby might exhibit a SASP-mediated immunosuppressive phenotype.

### Immunological characterization of TIS on TME of HCC

**Figure 7:**
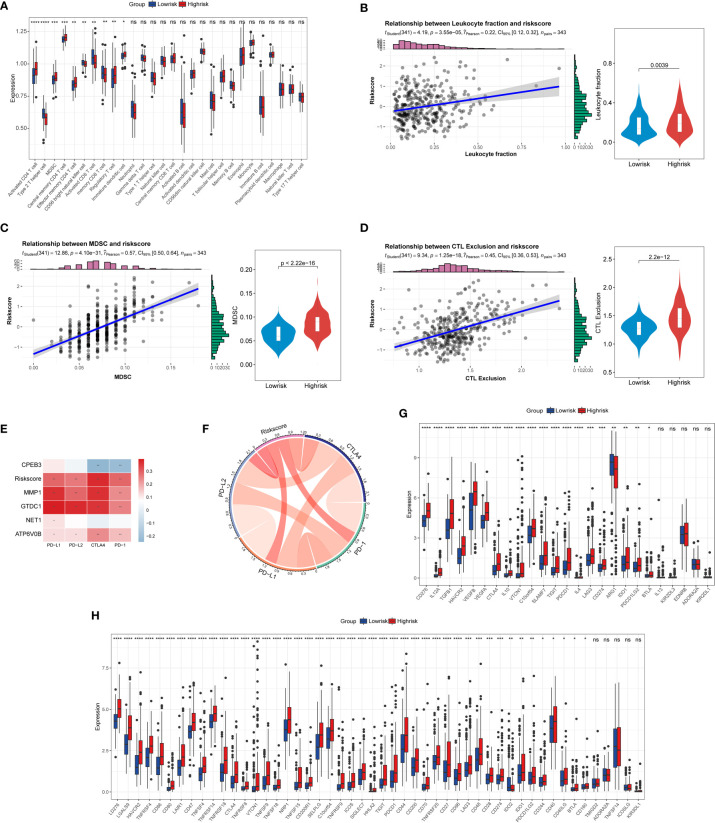
Correlation of TIS with immune infiltration, immune checkpoint genes, and immunomodulatory genes. **(A)** The boxplot for the alteration of 28 immune cells between the TIS-high and TIS-low groups. Differential leukocyte fraction **(B)**, MDSC **(C)**, and CTL exclusion score **(D)** between the TIS-high and TIS-low groups. Heatmap **(E)** and chordal graph **(F)** for the correlation of the common immune checkpoint genes, including PD1, CTLA4, PD-L1, and PD-L2, with TIS and TIS genes. The correlation of TIS with 25 immune checkpoint genes **(G)** and 50 immunomodulatory genes **(H)**; *P < 0.05; **P < 0.01; ***P < 0.001;****P < 0.0001; ns, no significance.

Previously published studies have corroborated that cellular senescence governs an irreplaceable role in the cancer immune-oncology context. Consequently, to dive deeper into the complex cross talk between TIS and tumor immunity, we first conducted ssGSEA to delineate the distinct landscape of infiltrated immune cells among TIS-high and TIS-low groups. An integrated boxplot of 28 immune cells showed a significant alteration in 10 cell proportions (**Figure 7A**). The TIS-high group exhibited a higher degree of immunosuppressive cells, including myeloid-derived suppressor cells (MDSC) and regulatory T cells, and decreased effector killing cells, including activated CD8 T cells and CD56 bright natural killer cells (**Figure 7A**). Interestingly, TIS-high also presented an increased distribution of activated CD4 T cells, including type 2 T helper cells and effector memory CD8 T cells. Additionally, a greater leukocyte fraction was observed in the high-risk group (**Figure 7B**). In parallel, the high levels of MDSC and CTL exclusion scores were captured in the high-risk group through the TIDE analysis platform (**Figures 7C, D**). Subsequently, we focused on the analysis of differential expression of immune checkpoint genes and immunomodulator genes between high-risk and low-risk groups. Our results emphasized that the TIS score as well as MMP, GTDC1, and ATP6V0B was statistically positively associated with PD1, CTLA4, PD-L1, and PD-L2, whereas CPEB3 was in negative concert with such immune checkpoint genes (**Figures 7E, F**). Beyond this, a significantly elevated expression of co-inhibitory immune checkpoint genes, including PDCD1, CTLA4, LAG3, TIGIT, and HAVCR2, which drive functional exhaustion of T cells, was captured in the TIS-high group (**Figure 7G**). An extended correlation analysis with regard to 50 immunomodulators deciphered that the vast majority of genes were remarkably corelated with TIS score (**Figure 7H**). Therefore, we speculated that the TIS-high subtype might present a tendency toward immuno-oncological inertia in HCC and might yield an unsatisfied response to immunotherapy.

### Predictive potential of TIS in immunotherapy response

**Figure 8:**
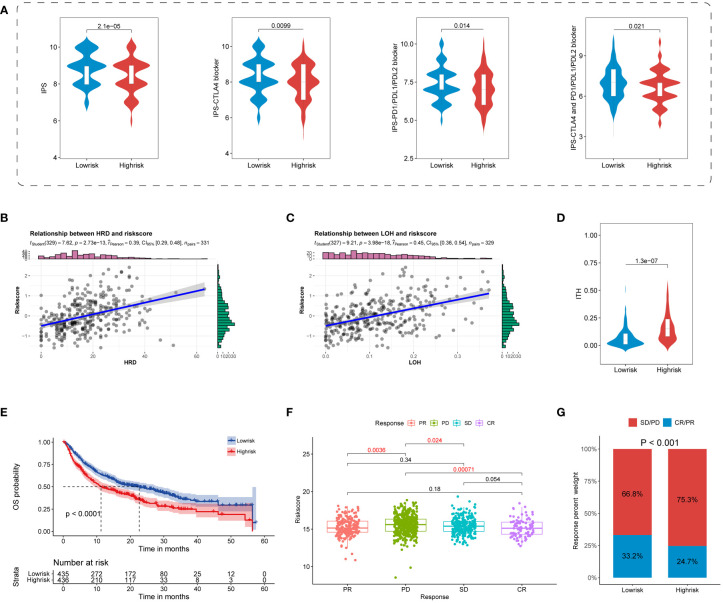
Predictive potential of TIS for immunotherapy response. Violin plots for the correlation of TIS with the four IPS **(A)** and ITH **(B)**. Scatterplots for the correlation of TIS with LOH **(C)** and HRD **(D)**. **(E)** The distinct prognostic pattern of TIS in immunotherapy data. Boxplot **(F)** and bar plot **(G)** for the differential immunotherapy response between the TIS-high and TIS-low groups. IPS, immunophenoscore; ITH, intratumor heterogeneity; LOH, loss of heterozygosity; HRD, homologous recombination deficiency.

Increasingly identified superior predictors, including IPS and ITH, were suggested to well predict and evaluate immunotherapy response (32, 51). Our results showed that all four types of IPS were primarily enriched in the TIS-low group, indicating a superior response to ICB in the TIS-low group (**Figure 8A**). Beyond this, TIS-high samples were featured with high ITH, indicating a poor immunotherapy response in the TIS-high group (**Figure 8B**). Considering that LOH of the HLA molecule was involved in immune escape, a scatter diagram of the TIS score and LOH was delineated. It was revealed that LOH was statistically positive correlated with TIS score (**Figure 8C**). Interestingly, elevated HRD, partially determined by LOH, was accompanied by increased TIS score, with higher HRD indicating a much improved immunotherapy response (**Figure 8D**). This finding was inconsistent with our hypothesis that TIS-high patients suffered an unsatisfied immunotherapy response. It was speculated that a set complex set of interaction mechanisms took responsibility for immunotherapy response, and a single biomarker was not sufficient for response prediction accuracy.

To convert theoretical prediction of TIS to real-world evidence, we extracted publicly available ICB cohorts of SKCM, KIRC, BLCA, STAD, HNSC, and GBM to further validate the practicability of TIS for predicting immunotherapy response. We discovered that patients in the TIS-low group distinctly yielded a prolonged OS compared with those assigned in the TIS-high group (**Figure 8E**, *P* < 0.0001). The enrolled patients treated with ICB exhibited differential response degrees defined as PD, SD, PR, and CR. As shown in the **Figure 8F**, patients’ evaluated CR presented the lowest TIS score, and patients’ evaluated PD exhibited the highest TIS score. Compared with the PD subgroup, patients in CR, PR, and SD were prone to a lower TIS score, with all *P* < 0.05. In other words, the patients with a low-TIS score exhibited increased susceptibility toward PR or CR, whereas patients with a high-TIS score were skewed toward PD or SD (**Figure 8G**, *P* < 0.001). These results evidenced that TIS could serve as a favorable predictor for immunotherapy and a lower TIS score might be in accordance with a better response to ICB therapy.

### Potential therapeutic value and candidate compound

**Figure 9:**
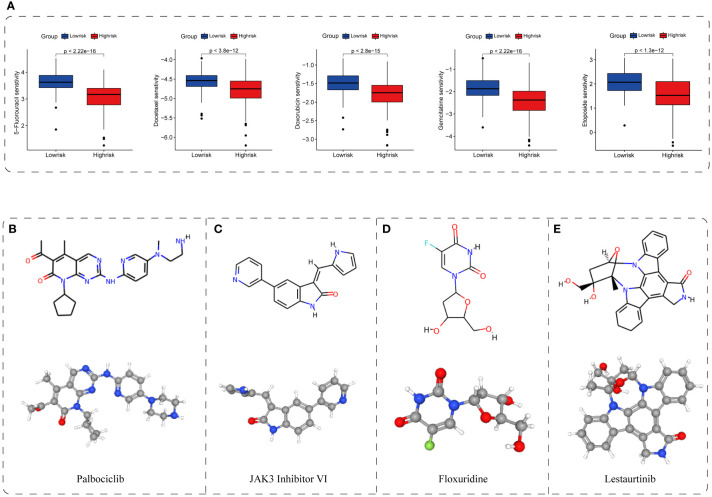
Application of TIS for drug sensitivity and putative compound. **(A)** The correlation between TIS and drug sensitivity. The 2D and 3D structures of the top four potential compounds including palbociclib **(B)**, JAK3 inhibitor VI **(C)**, floxuridine **(D)**, and lestaurtinib **(E)**.

To further determine the potential therapeutic value of the TIS classifier, we demonstrated the feasibility of identifying sensitive drug and candidate compounds through CGP and CMap databases (35, 36). We found that the high-risk group showed differential susceptibility to the majority of 212 drugs. Of these drugs, five conventional chemotherapeutic agents, namely, 5-fluorouracil, docetaxel, doxorubicin, gemcitabine, and etoposide, exhibited a lower normalized IC50 in the high-risk group, indicating a higher efficacy to high-risk patients (*P* < 0.001, **Figure 9A**). Additionally, small molecular inhibitors of several pathway targets involved in cellular senescence were permuted, covering cell-cycle inhibitors, bromodomain and extraterminal domain family (BET) inhibitors, PI3K-AKT pathway inhibitors, and multikinase inhibitors (4, 52–55). Patients in the TIS-high group presented higher susceptibility to these inhibitors, revealing the enriched cellular senescence in the TIS-high group (*P* < 0.001, **Figures S2A–D**). Additionally, we screened out and permuted the top four putative drugs (palbociclib, JAK3 inhibitor VI, floxuridine, and lestaurtinib) for TIS-high patients from 2,429 compounds, and the 2D and 3D structures of such four potential compounds are shown in **Figures 9B–E**. We also provided the information of 25 permuted compounds (**Supplementary Table S10**), and 32% (8/25) belong to cell-cycle inhibitors, indicating the favorable efficacy of cell-cycle inhibitors for TIS-high patients. These results suggested that TIS might provide a novel perspective for drug development and treatment selection.

### CPEB3 suppresses cell proliferation and invasion in HCC

**Figure 10:**
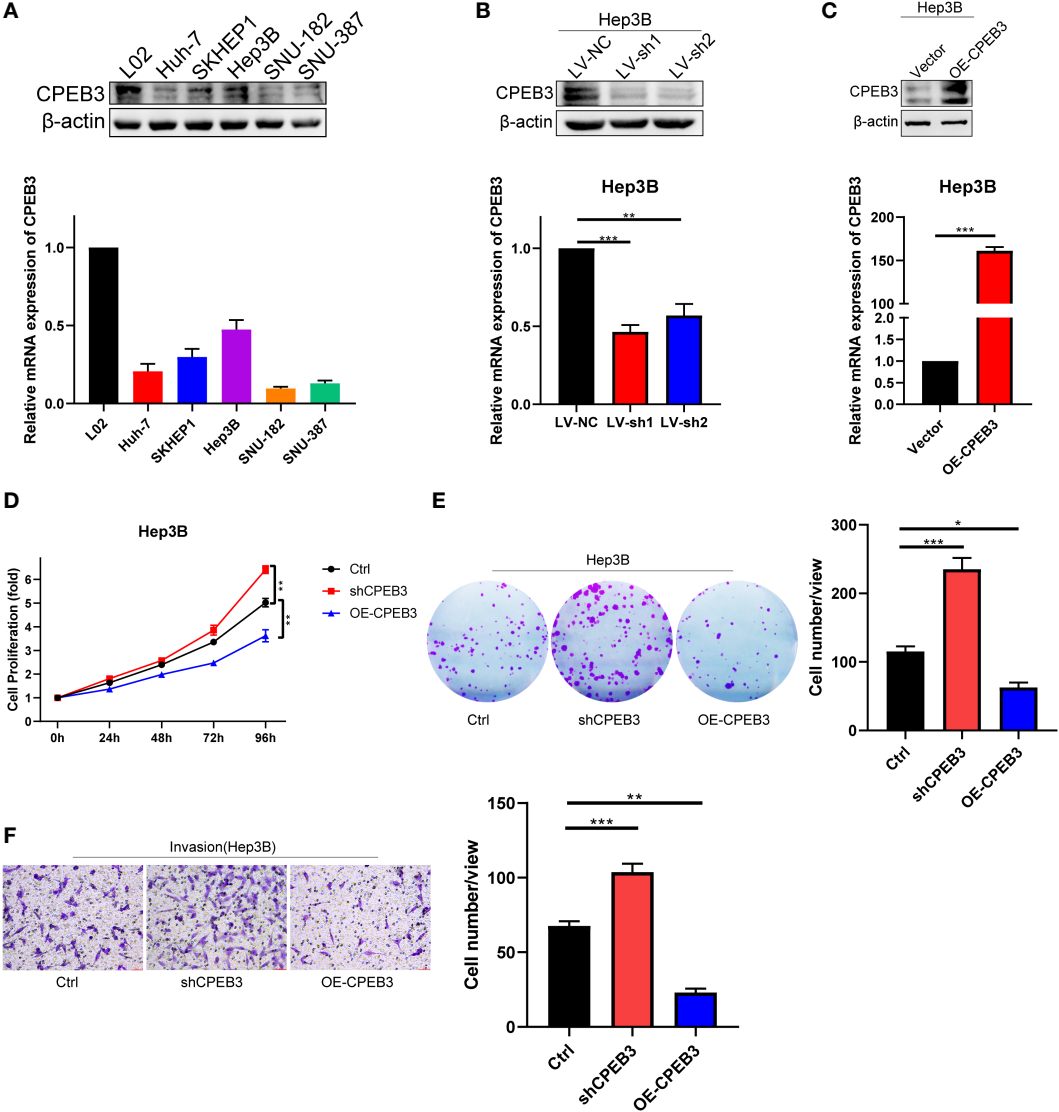
CPEB3 inhibits cell proliferation, colony formation, and invasion in HCC cells. **(A)** The CPEB3 abundance in normal liver cells and HCC cells at protein and mRNA levels. **(B)** Hep3B cell was stably transfected with LV-shNC and LV-shCPEB3. The expression of CPEB3 was detected using qRT-PCR and Western blot. **(C)** Hep3B cells were stably transfected with LV-ctrl and LV-CPEB3. The expression of CPEB3 in stably overexpressed and control Hep3B cells was detected using qRT-PCR and Western blot. **(D)** Cell Counting Kit-8 was used to quantify the proliferation of Hep3B-Ctrl, Hep3B-shCPEB3, and Hep3B-CPEB3. **(E)** Colonies of Hep3B-Ctrl, Hep3B-shCPEB3, and Hep3B-CPEB3 were quantified following continuous incubation for 14 days. **(F)** Transwell assays were carried out to determine the effect of CPEB3 on the cell invasion. *P < 0.05; **P < 0.01; ***P < 0.001.

To convert theoretical tumor biological behaviors of TIS genes to experimental evidence, we carried out experiments to reinforce our results. As aforementioned, CPEB3 was the only downregulated gene among TIS genes; in addition, CPEB3 has been documented to exhibit a striking downregulation across digestive tumor and function as a translational repressor and tumor suppressor according to the limited published studies (56–59). Therefore, CPEB3 was selected as the candidate gene for further validation. We first proceeded to validate the expression of CPEB3 in HCC cells and normal liver cells at protein and mRNA levels, and results demonstrated that CPEB3 was ubiquitously downregulated in HCC cells compared with L02 cells (**Figure 10A**). To characterize the effects of CPEB3 on the malignant phenotypes of HCC cells, we constructed stable CPEB3 knockdown- and overexpression-Hep3B cell lines (**Figures 10B, C**). It was revealed that CPEB3 knockdown enhanced Hep3B cell proliferation and CPEB3 overexpression attenuated Hep3B cell proliferation (**Figure 10D**). In addition, CPEB3 downregulation substantially boosted the clonogenicity ability and invasive capacity of Hep3B cells (**Figures 10E, F**). In contrast, Hep3B cells with overexpressed CPEB3 formed relatively less clones and inhibited cell-invasive ability (**Figures 10E, F**). These results indicated that CPEB3 serves as a negative regulator in the HCC setting and thereby attenuates malignant progression and metastasis.

## Discussion

Immunotherapy, especially ICB, is revolutionizing the therapeutic paradigm of HCC (3). Nevertheless, due to the inter- and intra-tumor heterogeneity, identification of a benefit subpopulation from immunotherapy remains a non-negligible barrier. Therefore, predictive biomarkers on immunotherapy response and prognosis are eagerly and urgently awaited to determine HCC subtypes and improve the personalized immunotherapy. To date, accumulating evidence has demonstrated that cellular senescence is involved in tumorigenesis and cancer progression and governs an indispensable role in TME through both cell autonomous and paracrine effects (4, 9, 60). However, how SASP-characterized cellular senescence interacts with tumor immune landscape and its potential in evaluating HCC prognosis, ICB response, and drug intervention are less well-established. Consequently, modeling HCC will pave a way for exploring the interaction of cellular senescence with the TME and deciphering the influential pattern of cellular senescence on HCC prognosis and immunotherapy response. Here, we attempted to screen out the HCC tumorigenic and immune infiltration-associated cellular senescence genes through multiple algorithms and thereby constructed an independent prognostic and immune-related signature named TIS incorporating five cellular senescence genes. Then, TIS was subsequently employed to dichotomize HCC patients into high- and low-risk subgroups in both the training and validation cohorts. As expected, TIS-high patients turned out to have a higher mortality exposure and were accompanied by immunosuppressive cell infiltration and effector killing cell exclusion. Markedly, a ubiquitously elevated expression of co-inhibitory immune checkpoint genes was identified in the TIS-high patients, who exhibited a worse response to ICB immunotherapy. Compared with the TIS-low group, the TIS-high group was characterized by the activation of carcinogenetic pathways, such as G2M checkpoint, MYC, EMT, and PI3K-AKT-mTOR pathway, and subsequently, potential compounds targeting TIS were ultimately determined *via* the CMap database. The present study engaged an integrative analysis to achieve a deeper and comprehensive understanding of cellular senescence and represented innovative exploration and application for signature construction with tumorigenic and immune infiltration-associated senescence genes, whose superior predictive performance was identified in terms of HCC prognosis, immune infiltration and evasion, immunotherapy response, and even putative drug identification.

Chronic liver diseases, such as non-alcoholic fatty liver, non-alcoholic steatohepatitis, and cirrhosis, are recognized prodromes of HCC and are accompanied by a process of hepatocellular senescence (5). Additionally, there is no definite tumorigenic and immune infiltration-associated senescence gene set available. Consequently, we performed WGCNA and ESTIMATE algorithms, for the first time, to determine the specific tumor- and immune infiltration-associated senescence genes. Subsequently, a novel prognostic signature (TIS) consisting of five senescence genes, namely, NET1, ATP6V0B, MMP1, GTDC1, and CPEB3, was constructed through LASSO, KM, and Cox regression algorithms. Of these TIS genes, only CPEB3 exhibited a decreased expression in both the TIS-high group and paired tumor samples, and an abnormally decreased CPEB3 expression conferred a comparatively worse prognosis, indicating the protective role of CPEB3. In contrast to CPEB3, the remaining TIS genes, including NET1, ATP6V0B, MMP1, and GTDC1, govern a detrimental role in HCC. For instance, neuroepithelial cell transforming gene 1 (NET1) is documented to promote hepatocarcinogenesis and metastasis through the PI3K/AKT pathway (61). To date, increasing HCC signatures have been documented to classify clusters and predict prognosis, such as the TP-53-associated four-gene signature by Long (45), ferroptosis-associated 10-gene signature by Liang (43), immune-associated nine-gene signature by Wang (42), and hypoxia-associated four-gene signature by Zeng (41). Markedly, our five-gene TIS model yielded favorable prognostic performance with a higher AUC than most previous signatures (40–45). Moreover, unlike the previously mentioned literatures only limitedly delineating prognostic signature characterization, our study integratively displayed the landscape of HCC prognosis, TME, and potential targeting drugs, which was infrequently reported. To sum up, TIS exhibited the potential to mirror HCC prognosis and could serve as an effective classifier for HCC.

Subsequently, through uncovering the differential underlying mechanisms between TIS-high and TIS-low groups, we found that the most carcinogenetic pathways were overactivated in the TIS-high group, covering GLYCOLYSIS, DNA repair, G2M checkpoint, MYC, and EMT signaling pathways. Notably, all of the mentioned pathways were documented to give rise to immunosuppression or weak immunotherapy response according to the public literatures (62–65). Additionally, abnormal metabolic reprograming in TIS-high was characterized by activated glycolysis and suppressed oxidative phosphorylation, which aggravate acidosis, hypoxia, angiogenesis, EMT, and immunosuppression, thereby promoting the malignant phenotypes of invasion and metastasis (66, 67). We also delineated the SASP landscape between the TIS-high and TIS-low groups. Our analyzed results revealed that the TIS-high group exhibited a significant elevated expression of multiple SASP, including interleukin: IL-1A and IL-1B; proteases: MMP4, MMP1, MMP 12, and MMP14; growth factors: IGFBP, VEGF and ANG; receptors: ICAMs; and chemokine: CXCL3, CXCL8, and CXCL5. These altered SASP might contribute to a malignant phenotype and govern a vital role in TME reshaping, which ultimately leads to immune evasion and tumor development (9, 68–70).

Currently, no definite conclusion has been drawn on whether and how cellular senescence modulates immune infiltration and immunomodulators and thereby affects the therapeutic response to ICB. On the basis of our results, the TIS score exhibited an inverse association with effector killing cells, such as activated CD8 T cells and CD56 bright natural killer cells, whereas it was positively implicated with immunosuppressive cells, such as MDSC and regulatory T cells, indicating that patients with a higher TIS score are vulnerable to immunosuppression and attenuated tumor clearance. Indeed, MDSC could release SASP-MMPs to facilitate tumor cell invasion through directly augmenting angiogenesis and lymphangiogenesis (71, 72). Interestingly, upregulated abundance of activated CD4 T cells, which was commonly considered as an antitumor effector (73, 74), was detected in our results. We speculated that the antitumor capacity of activated CD4 T cells may be cloaked and limited by senescence phenotype and immunosuppressed molecules on tumor cells (such as PD-L1). We also uncovered the ubiquitous correlation of the TIS score with the majority of 50 common immune checkpoint genes, containing PD-1, CTLA4, PDL1, and the biomarkers of T-cell exhaustion, which are associated with T-cell-mediated immunotherapy (3, 34). As aforementioned, IPS and ITH were documented as superior indictors for immunotherapeutic response, with a higher IPS or lower ITH score representing a favorable response to ICB (32, 51). In our results, the TIS-high group was accompanied by a higher score of IPS and a lower score of ITH, indicating a theoretically less response to ICB. Such predictive performance of TIS was subsequently proved in real-world ICB data of solid tumors. The TIS-high group exhibited a higher proportion of PD and SD, suggesting the unsatisfied response to ICB. Collectively, TIS showed great potential to serve as a substantial integrative predictor for immune infiltration and evasion and immunotherapeutic response to ICB.

As another application of our TIS classifier, we demonstrated the feasibility of identifying sensitive drug and candidate compounds through CGP and CMap databases (35, 36). Through matching the up- and downregulated genes with the drug-treated RNA-sequencing data, we detected that the TIS-high group showed susceptibility to several conventional chemotherapeutic agents, including 5-fluorouracil, docetaxel, doxorubicin, gemcitabine, and etoposide, uncovering the specific therapeutic potential of such conventional agents for cellular senescence. Beyond this, we also identified the four candidate compounds with the most potential from a total of 2,429 compounds. Among them, palbociclib, the most pro-senescence relevant CDK4/6 inhibitor, has been documented with superior antitumor capability across HCC, melanoma, and breast cancer, through inducing a senescence phenotype (75, 76). However, in the late stage of palbociclib treatment, enhanced HCC progression was observed in an experimental Fah^-/-^ mouse model of HCC (5). It was speculated that early hepatocellular senescence could provoke immune activation to eliminate tumor cells and senescent cells. With the accumulation of senescent hepatocellular and SASP secretion, the tumor microenvironmental context was reshaped and thereby contributed to HCC progression, which might partially describe the drug resistance mechanism of senescence-targeting drugs. Consequently, elimination of accumulated detrimental senescence and induction of acute cellular senescence might be an important direction, and more studies on the interaction between cellular senescence and antitumor microenvironment are warranted. Noticeably, the remaining three compounds have also been reported to have antitumor capability, although their correlation with cellular senescence has not been characterized (77–79). In consideration of the complicated involved pathways in cellular senescence (4, 52–55), we permuted additional candidate compounds, including cell-cycle inhibitors, bromodomain and BET inhibitors, PI3K-AKT pathway inhibitors, and multikinase inhibitors, and the TIS-high group exhibited a higher susceptibility to these drugs. These candidate compounds shed light on the HCC therapeutic strategy especially for the patients in the high-risk subgroup, and more in-depth explorations are warranted to provide insight into the interaction mechanisms of these small compounds with cellular senescence.

To further definite the outsized oncological role of TIS genes, we conducted *in vitro* experimental validation and delineated the association between CPEB3 and HCC phenotypes. In accordance with our anticipation, CPEB3 knockdown promoted Hep3B proliferation, clonogenicity, and invasion, whereas CPEB3 overexpression attenuated such phenotypes, indicating the definite role of CPEB3 in tumorigenesis and development. Further, more research is required to determine the senescence oncology role of TIS genes and to delineate the interaction of TIS genes with the underlying pathways, such as G2M checkpoint, MYC, EMT, and PI3K-AKT-mTOR pathways.

## Conclusion

All the analyses taken together, we established a tumorigenic and immune infiltration-associated TIS model for the predictions of HCC prognosis and immunotherapy efficacy based on a senescence gene-guided strategy, and its performance was well-verified by external transcriptome data and immunotherapy data. Through characterizing the complex linkage of TIS with oncogenic pathways and SASP, our results provided insight into the underlying mechanisms of TIS on tumorigenesis and progression as well as TME reshaping. Combining these results and the interplay between TIS and immune infiltration, immune checkpoint factors, and other biomarkers, we demonstrated that TIS could effectively discriminate responders and non-responders to enable a more precise benefit stratification of ICB therapy. Additionally, we identified several potentially senescence-related candidate compounds as an alternative strategy for HCC treatment especially for the patients in the high-risk subgroup. Therefore, this work might facilitate prognostic biomarker identification and provide certain guiding significance for personalized immunotherapy.

## Data availability statement

The datasets presented in this study can be found in online repositories. The names of the repository/repositories and accession number(s) can be found in the article/**Supplementary Material**.

## Author contributions

YL and FT contributed to the conception and design of the study. YL performed the literature review, data analysis,and manuscript drafting. FT made critical revisions and was responsible for the final manuscript. HL and HF participated in data analysis. G-SD was the supervisor and participated in the manuscript editing. All authors contributed to the article and approved the submitted version.

## Funding

This article was supported by national Natural Science Foundation of China, No.81702923 and No. 81871262.

## Conflict of interest

The authors declare that the research was conducted in the absence of any commercial or financial relationships that could be construed as a potential conflict of interest.

## Publisher’s note

All claims expressed in this article are solely those of the authors and do not necessarily represent those of their affiliated organizations, or those of the publisher, the editors and the reviewers. Any product that may be evaluated in this article, or claim that may be made by its manufacturer, is not guaranteed or endorsed by the publisher.

## Supplementary material

Supplementary Table 1 List of raw senecence genes.

Supplementary Table 2 Oligonucleotides used in this study.

Supplementary Table 3 Primers used in this study.

Supplementary Table 4 Antibodies used in this study.

Supplementary Table 5 List of immune infiltration genes.

Supplementary Table 6 List of WGCNA genes.

Supplementary Table 7 The 127 intersection-prognostic genes of KM- and Cox-analyses results with *P* < 0.05.

Supplementary Table 8 The GSVA results regarding the TIS risk groups (High *vs*. Low).

Supplementary Table 9 The GSEA results regarding the TIS risk groups (High *vs*. Low).

Supplementary Table 10 The top 25 potential therapeutic compounds.

Supplementary Figure 1 Distinct SASP landscapes between the TIS-high and TIS-low groups. Differential expression of five types of SASP factors, including interleukins **(A)**, soluble or shed receptors or ligands **(B)**, proteases and regulators **(C)**, chemokines **(D)**, and growth factors and regulators **(E)**, between TIS-high and TIS-low groups.

Supplementary Figure 2 The relationship of TIS with the drug sensitivity of four senescence-related pathway inhibitors. The distinct drug sensitivity of cell-cycle inhibitors **(A)**, BET inhibitors **(B)**, PI3K-AKT pathway inhibitors **(C)**, and multikinase inhibitors **(D)**. BET: bromodomain and extraterminal domain family.
