# Supplementary material for: A cellular senescence-related classifier based on a tumorigenesis- and immune infiltration-guided strategy can predict prognosis, immunotherapy response, and candidate drugs in hepatocellular carcinoma
Source: Front Immunol. 2022 Nov 15;13:974377. doi: 10.3389/fimmu.2022.974377 (PMC9705748; doi:10.3389/fimmu.2022.974377)
Supplement: Supplementary Table 1 — List of raw senecence genes. [file DataSheet_1.zip › Supplementary Materials/Supplementary Table 10. The top 25 potential therapeutic compounds.docx]

**Table S10. The top 25 potential therapeutic compounds**

| Score | Compound name | Description |
| --- | --- | --- |
| -99.72 | Palbociclib | CDK inhibitor |
| -99.51 | JAK3-inhibitor-VI | JAK inhibitor |
| -99.19 | Floxuridine | DNA synthesis inhibitor |
| -99.08 | Lestaurtinib | FLT3 inhibitor |
| -99.01 | HG-5-113-01 | Protein kinase inhibitor |
| -98.91 | Purvalanol-a | CDK inhibitor |
| -98.45 | CGP-60474 | CDK inhibitor |
| -98.41 | Ochratoxin-a | Phenylalanyl tRNA synthetase inhibitor |
| -98.27 | BMS-345541 | IKK inhibitor |
| -98.27 | ER-27319 | Mediator release inhibitor |
| -98.27 | Alvocidib | CDK inhibitor |
| -98.24 | PHA-793887 | CDK inhibitor |
| -98.24 | PF-562271 | Focal adhesion kinase inhibitor |
| -98.24 | ZG-10 | JNK inhibitor |
| -98.24 | JNK-9L | JNK inhibitor |
| -98.24 | JNJ-7706621 | CDK inhibitor |
| -98.2 | Dactinomycin | RNA polymerase inhibitor |
| -98.2 | Camptothecin | Topoisomerase inhibitor |
| -98.2 | Pidorubicine | Topoisomerase inhibitor |
| -98.17 | AT-7519 | CDK inhibitor |
| -98.1 | Bisindolylmaleimide-ix | CDK inhibitor |
| -98.06 | Triptolide | RNA polymerase inhibitor |
| -98.03 | Cyclopamine | Smoothened receptor antagonist |
| -98 | Linifanib | PDGFR receptor inhibitor |
| -97.99 | 5-iodotubercidin | Adenosine kinase inhibitor |
