# Supplementary material for: A cellular senescence-related classifier based on a tumorigenesis- and immune infiltration-guided strategy can predict prognosis, immunotherapy response, and candidate drugs in hepatocellular carcinoma
Source: Front Immunol. 2022 Nov 15;13:974377. doi: 10.3389/fimmu.2022.974377 (PMC9705748; doi:10.3389/fimmu.2022.974377)
Supplement: Supplementary Table 1 — List of raw senecence genes. [file DataSheet_1.zip › Supplementary Materials/Supplementary Table 2. Oligonucleotides used in this study.docx]

**Table S2. Oligonucleotides used in this study**

| Name | Sequence (5’-3’) |
| --- | --- |
| pLKO.1-sh1 | CCGGGCTGATATAATGTGGAGGAATCTCGAGATTCCTCCACATTATATCAGCTTTTTT |
| pLKO.1-sh2 | CCGGGCTGATATAATGTGGAGGAATCTCGAGATTCCTCCACATTATATCAGCTTTTTT |
| pLKO.1-shNC | CCGGCCGCAGGTATGCACGCGTCTCGAGACGCGTGCATACCTGCGGTTTTTT |
