# Supplementary material for: A cellular senescence-related classifier based on a tumorigenesis- and immune infiltration-guided strategy can predict prognosis, immunotherapy response, and candidate drugs in hepatocellular carcinoma
Source: Front Immunol. 2022 Nov 15;13:974377. doi: 10.3389/fimmu.2022.974377 (PMC9705748; doi:10.3389/fimmu.2022.974377)
Supplement: Supplementary Table 1 — List of raw senecence genes. [file DataSheet_1.zip › Supplementary Materials/Supplementary Table 3. Primers used in this study.docx]

**Table S3. Primers used in this study**

| Name | Sequence (5’-3’) |
| --- | --- |
| CPEB3 | F: GAGTCCAGCGTATCCGAAGC |
|  | R: GAGCGGTGATTCCATCTGCAT |
| β-actin | F: CCTGGCACCCAGCACAAT |
|  | R: GGGCCGGACTCGTCATAC |
