# Supplementary material for: A cellular senescence-related classifier based on a tumorigenesis- and immune infiltration-guided strategy can predict prognosis, immunotherapy response, and candidate drugs in hepatocellular carcinoma
Source: Front Immunol. 2022 Nov 15;13:974377. doi: 10.3389/fimmu.2022.974377 (PMC9705748; doi:10.3389/fimmu.2022.974377)
Supplement: Supplementary Table 1 — List of raw senecence genes. [file DataSheet_1.zip › Supplementary Materials/Supplementary Table 4. Antibodies used in this study.docx]

**Table S4. Antibodies used in this study**

| Target | Species | Dilution | Manufacture |
| --- | --- | --- | --- |
| CPEB3 | Rabbit | 1: 1000 | Bioss, bs-11340R |
| β-actin | Rabbit | 1: 5000 | SIMUWU, SD0034 |
| IgG (HRP) | Goat | 1: 4000 | Proteintech, SA00001-2 |
