# Supplementary material for: A cellular senescence-related classifier based on a tumorigenesis- and immune infiltration-guided strategy can predict prognosis, immunotherapy response, and candidate drugs in hepatocellular carcinoma
Source: Front Immunol. 2022 Nov 15;13:974377. doi: 10.3389/fimmu.2022.974377 (PMC9705748; doi:10.3389/fimmu.2022.974377)
Supplement: Supplementary Table 1 — List of raw senecence genes. [file DataSheet_1.zip › Supplementary Materials/Supplementary Table 7. The 127 intersection-prognostic genes of KM- and Cox-analyses results with P 0.05.docx]

**Table S7. The 127 intersection-prognostic genes of KM- and Cox-analyses results with *P* < 0.05**

| Genes | HR | HR_lower | HR_upper | *P*-value |
| --- | --- | --- | --- | --- |
| NET1 | 1.598 | 1.25 | 2.043 | 0 |
| ATP6V0B | 1.912 | 1.464 | 2.496 | 0 |
| MMP1 | 1.406 | 1.246 | 1.586 | 0 |
| GTDC1 | 2.571 | 1.761 | 3.752 | 0 |
| CPEB3 | 0.724 | 0.583 | 0.9 | 0.004 |
| LPCAT1 | 1.495 | 1.308 | 1.708 | 0 |
| YBX1 | 2.33 | 1.796 | 3.021 | 0 |
| KIAA0930 | 1.736 | 1.417 | 2.125 | 0 |
| ANXA5 | 1.416 | 1.202 | 1.667 | 0 |
| ATP1B3 | 1.436 | 1.231 | 1.675 | 0 |
| PGM2L1 | 1.386 | 1.102 | 1.742 | 0.005 |
| SPP1 | 1.134 | 1.076 | 1.196 | 0 |
| PPP1R14B | 1.481 | 1.221 | 1.797 | 0 |
| SLC2A2 | 0.865 | 0.803 | 0.932 | 0 |
| CORO7 | 1.425 | 1.091 | 1.86 | 0.009 |
| CDKN2D | 1.547 | 1.245 | 1.923 | 0 |
| CDC25B | 1.479 | 1.259 | 1.738 | 0 |
| OAZ1 | 1.543 | 1.203 | 1.98 | 0.001 |
| MARCKSL1 | 1.372 | 1.195 | 1.576 | 0 |
| ABCC1 | 1.323 | 1.153 | 1.519 | 0 |
| SNHG12 | 1.448 | 1.17 | 1.793 | 0.001 |
| IKBIP | 1.604 | 1.299 | 1.98 | 0 |
| HOMER3 | 1.278 | 1.116 | 1.463 | 0 |
| SLC2A1 | 1.437 | 1.266 | 1.63 | 0 |
| PFKP | 1.221 | 1.106 | 1.348 | 0 |
| ENO2 | 1.223 | 1.085 | 1.379 | 0.001 |
| VOPP1 | 1.531 | 1.223 | 1.915 | 0 |
| IER3 | 1.2 | 1.077 | 1.336 | 0.001 |
| PSMA5 | 1.799 | 1.331 | 2.43 | 0 |
| CLIC1 | 1.395 | 1.181 | 1.646 | 0 |
| VASP | 1.447 | 1.186 | 1.766 | 0 |
| RWDD1 | 1.69 | 1.232 | 2.318 | 0.001 |
| CMTM7 | 1.434 | 1.187 | 1.732 | 0 |
| CRHBP | 0.828 | 0.706 | 0.973 | 0.021 |
| NUDT1 | 1.358 | 1.16 | 1.591 | 0 |
| YWHAZ | 1.515 | 1.24 | 1.852 | 0 |
| CTHRC1 | 1.199 | 1.089 | 1.321 | 0 |
| RPS6KA4 | 1.613 | 1.201 | 2.166 | 0.001 |
| ALDOA | 1.391 | 1.207 | 1.603 | 0 |
| PKIB | 1.265 | 1.134 | 1.411 | 0 |
| PGF | 1.266 | 1.096 | 1.462 | 0.001 |
| ABAT | 0.872 | 0.786 | 0.969 | 0.011 |
| SIRT6 | 1.688 | 1.282 | 2.223 | 0 |
| PRKCD | 1.525 | 1.266 | 1.838 | 0 |
| ARPC2 | 1.751 | 1.331 | 2.304 | 0 |
| ARPC1B | 1.304 | 1.092 | 1.558 | 0.003 |
| ECI2 | 0.779 | 0.627 | 0.968 | 0.024 |
| MTHFD1L | 1.578 | 1.294 | 1.924 | 0 |
| POLE4 | 1.515 | 1.226 | 1.871 | 0 |
| ACER3 | 1.512 | 1.176 | 1.945 | 0.001 |
| BAK1 | 1.396 | 1.173 | 1.662 | 0 |
| NFKB2 | 1.285 | 1.034 | 1.597 | 0.023 |
| APOC3 | 0.919 | 0.859 | 0.984 | 0.015 |
| CXCL1 | 1.116 | 1.039 | 1.2 | 0.003 |
| C15orf48 | 1.161 | 1.062 | 1.27 | 0.001 |
| TMSB10 | 1.179 | 1.056 | 1.316 | 0.003 |
| MAPK3 | 1.625 | 1.24 | 2.129 | 0 |
| SERPINB6 | 1.272 | 1.016 | 1.594 | 0.036 |
| OXCT1 | 1.241 | 1.092 | 1.41 | 0.001 |
| TMEM263 | 1.315 | 1.026 | 1.685 | 0.031 |
| CHPF2 | 1.698 | 1.301 | 2.216 | 0 |
| DRAM1 | 1.265 | 1.059 | 1.511 | 0.01 |
| AASS | 0.874 | 0.776 | 0.985 | 0.027 |
| CXCL8 | 1.153 | 1.065 | 1.249 | 0 |
| CDO1 | 0.889 | 0.821 | 0.962 | 0.003 |
| UCHL1 | 1.135 | 1.053 | 1.224 | 0.001 |
| RGN | 0.832 | 0.746 | 0.927 | 0.001 |
| DRAP1 | 1.87 | 1.356 | 2.58 | 0 |
| BLOC1S4 | 1.735 | 1.326 | 2.269 | 0 |
| CPB2 | 0.863 | 0.789 | 0.944 | 0.001 |
| EZR | 1.237 | 1.057 | 1.448 | 0.008 |
| TM4SF1 | 1.134 | 1.001 | 1.286 | 0.049 |
| AIP | 1.412 | 1.052 | 1.894 | 0.022 |
| MMP7 | 1.118 | 1.039 | 1.203 | 0.003 |
| TAGLN2 | 1.482 | 1.211 | 1.812 | 0 |
| ADH6 | 0.894 | 0.808 | 0.99 | 0.031 |
| CIB2 | 1.222 | 1.063 | 1.405 | 0.005 |
| SHISA5 | 1.767 | 1.29 | 2.42 | 0 |
| EIF2AK1 | 1.655 | 1.151 | 2.38 | 0.007 |
| VKORC1L1 | 1.566 | 1.16 | 2.113 | 0.003 |
| TOR4A | 1.383 | 1.188 | 1.61 | 0 |
| TRAFD1 | 1.351 | 1.063 | 1.717 | 0.014 |
| RBM3 | 1.315 | 1.049 | 1.647 | 0.017 |
| SLC1A2 | 0.882 | 0.799 | 0.973 | 0.012 |
| ITPR3 | 1.199 | 1.065 | 1.349 | 0.003 |
| HIF1A | 1.242 | 1.075 | 1.435 | 0.003 |
| NPC2 | 1.387 | 1.128 | 1.706 | 0.002 |
| SLC12A2 | 1.211 | 1.054 | 1.393 | 0.007 |
| TUBA4A | 1.373 | 1.17 | 1.612 | 0 |
| C15orf39 | 1.311 | 1.085 | 1.584 | 0.005 |
| ACTB | 1.505 | 1.129 | 2.005 | 0.005 |
| CAPNS1 | 1.31 | 1.02 | 1.684 | 0.035 |
| ALDH6A1 | 0.881 | 0.777 | 0.998 | 0.047 |
| PARP12 | 1.485 | 1.158 | 1.905 | 0.002 |
| ELF4 | 1.25 | 1.082 | 1.444 | 0.003 |
| KNG1 | 0.891 | 0.828 | 0.959 | 0.002 |
| PTPRE | 1.252 | 1.021 | 1.537 | 0.031 |
| SLC6A6 | 1.198 | 1.055 | 1.361 | 0.005 |
| PPP1R9B | 1.225 | 1.004 | 1.495 | 0.046 |
| RBP4 | 0.86 | 0.789 | 0.938 | 0.001 |
| BAX | 1.33 | 1.068 | 1.655 | 0.011 |
| CTH | 0.9 | 0.819 | 0.988 | 0.027 |
| LRPPRC | 1.787 | 1.327 | 2.408 | 0 |
| CTTNBP2NL | 1.309 | 1.063 | 1.611 | 0.011 |
| CLK2 | 1.365 | 1.08 | 1.726 | 0.009 |
| IFI27L2 | 1.192 | 1.051 | 1.351 | 0.006 |
| MICALL2 | 1.265 | 1.049 | 1.527 | 0.014 |
| SLC6A1 | 0.873 | 0.795 | 0.957 | 0.004 |
| PFN1 | 1.504 | 1.157 | 1.954 | 0.002 |
| KLF9 | 0.836 | 0.729 | 0.96 | 0.011 |
| ATP8B2 | 1.248 | 1.054 | 1.477 | 0.01 |
| CAT | 0.85 | 0.725 | 0.996 | 0.045 |
| PSMA3 | 2.013 | 1.388 | 2.918 | 0 |
| CCL26 | 1.168 | 1.015 | 1.345 | 0.03 |
| BRAF | 1.428 | 1.063 | 1.918 | 0.018 |
| HSPB8 | 1.104 | 1.006 | 1.211 | 0.037 |
| TMED3 | 1.169 | 1.021 | 1.339 | 0.024 |
| SERPINH1 | 1.274 | 1.085 | 1.496 | 0.003 |
| ASAP1 | 1.468 | 1.219 | 1.767 | 0 |
| ANXA2 | 1.359 | 1.159 | 1.593 | 0 |
| ZKSCAN3 | 1.265 | 1.027 | 1.56 | 0.027 |
| NMT1 | 1.423 | 1.044 | 1.94 | 0.026 |
| TGFB1 | 1.178 | 1.041 | 1.333 | 0.009 |
| DBN1 | 1.218 | 1.078 | 1.377 | 0.002 |
| IFNAR2 | 1.41 | 1.064 | 1.868 | 0.017 |
| QPCT | 1.153 | 1.022 | 1.3 | 0.02 |
| ADAMTS3 | 1.632 | 1.168 | 2.281 | 0.004 |
