# Supplementary material for: A cellular senescence-related classifier based on a tumorigenesis- and immune infiltration-guided strategy can predict prognosis, immunotherapy response, and candidate drugs in hepatocellular carcinoma
Source: Front Immunol. 2022 Nov 15;13:974377. doi: 10.3389/fimmu.2022.974377 (PMC9705748; doi:10.3389/fimmu.2022.974377)
Supplement: Supplementary Table 1 — List of raw senecence genes. [file DataSheet_1.zip › Supplementary Materials/Supplementary Table 8. The GSVA results regarding the TIS risk groups (High vs. Low).docx]

**Table S8. The GSVA results regarding the TIS risk groups (High vs. Low)**

| Pathways | t-value | *P*-value | Adjust p-value |
| --- | --- | --- | --- |
| BILE_ACID_METABOLISM | -9.198 | 3.56E-18 | 3.56E-17 |
| FATTY_ACID_METABOLISM | -8.921 | 2.77E-17 | 1.98E-16 |
| XENOBIOTIC_METABOLISM | -8.176 | 5.67E-15 | 3.15E-14 |
| OXIDATIVE_PHOSPHORYLATION | -7.562 | 3.65E-13 | 1.66E-12 |
| PEROXISOME | -6.832 | 3.80E-11 | 1.27E-10 |
| ADIPOGENESIS | -5.971 | 5.86E-09 | 1.72E-08 |
| KRAS_SIGNALING_DN | -5.649 | 3.39E-08 | 9.41E-08 |
| COAGULATION | -3.085 | 0.002200689 | 0.003334377 |
| HEME_METABOLISM | -3.009 | 0.002812222 | 0.00401746 |
| PANCREAS_BETA_CELLS | -2.124 | 0.034398529 | 0.041949426 |
| KRAS_SIGNALING_UP | 2.049 | 0.041218823 | 0.049070027 |
| COMPLEMENT | 2.175 | 0.030304634 | 0.037880793 |
| APICAL_SURFACE | 2.308 | 0.021576722 | 0.027662464 |
| PROTEIN_SECRETION | 2.514 | 0.012395186 | 0.016309455 |
| TNFA_SIGNALING_VIA_NFKB | 2.924 | 0.003684778 | 0.00497943 |
| IL6_JAK_STAT3_SIGNALING | 2.940 | 0.003501988 | 0.004863873 |
| ALLOGRAFT_REJECTION | 3.058 | 0.002400767 | 0.003530539 |
| TGF_BETA_SIGNALING | 3.149 | 0.001781922 | 0.002784254 |
| APOPTOSIS | 3.158 | 0.001727601 | 0.002784254 |
| WNT_BETA_CATENIN_SIGNALING | 3.214 | 0.001431615 | 0.002386025 |
| INFLAMMATORY_RESPONSE | 3.364 | 0.000855195 | 0.001474475 |
| HEDGEHOG_SIGNALING | 3.382 | 0.000803701 | 0.001435181 |
| ANGIOGENESIS | 3.456 | 0.000617398 | 0.00114333 |
| NOTCH_SIGNALING | 3.482 | 0.0005617 | 0.001080193 |
| P53_PATHWAY | 3.659 | 0.000293271 | 0.000586543 |
| IL2_STAT5_SIGNALING | 3.697 | 0.000253894 | 0.000528945 |
| HYPOXIA | 3.792 | 0.000176118 | 0.000382864 |
| EPITHELIAL_MESENCHYMAL_TRANSITION | 4.062 | 6.02E-05 | 0.000136798 |
| MYC_TARGETS_V2 | 4.313 | 2.11E-05 | 5.02E-05 |
| APICAL_JUNCTION | 4.950 | 1.16E-06 | 2.91E-06 |
| ESTROGEN_RESPONSE_LATE | 5.451 | 9.57E-08 | 2.52E-07 |
| UNFOLDED_PROTEIN_RESPONSE | 6.753 | 6.18E-11 | 1.93E-10 |
| PI3K_AKT_MTOR_SIGNALING | 6.886 | 2.73E-11 | 9.73E-11 |
| UV_RESPONSE_UP | 7.008 | 1.28E-11 | 4.91E-11 |
| DNA_REPAIR | 7.184 | 4.22E-12 | 1.76E-11 |
| MTORC1_SIGNALING | 7.657 | 1.95E-13 | 9.73E-13 |
| GLYCOLYSIS | 8.211 | 4.45E-15 | 2.78E-14 |
| SPERMATOGENESIS | 9.152 | 5.04E-18 | 4.20E-17 |
| MITOTIC_SPINDLE | 10.426 | 2.66E-22 | 3.33E-21 |
| E2F_TARGETS | 11.081 | 1.31E-24 | 2.18E-23 |
| MYC_TARGETS_V1 | 11.464 | 5.44E-26 | 1.36E-24 |
| G2M_CHECKPOINT | 12.074 | 3.14E-28 | 1.57E-26 |
