# Supplementary material for: A cellular senescence-related classifier based on a tumorigenesis- and immune infiltration-guided strategy can predict prognosis, immunotherapy response, and candidate drugs in hepatocellular carcinoma
Source: Front Immunol. 2022 Nov 15;13:974377. doi: 10.3389/fimmu.2022.974377 (PMC9705748; doi:10.3389/fimmu.2022.974377)
Supplement: Supplementary Table 1 — List of raw senecence genes. [file DataSheet_1.zip › Supplementary Materials/Supplementary Table 9. The GSEA results regarding the TIS risk groups (High vs. Low).docx]

**Table S9. The GSEA results regarding the TIS risk groups (High vs. Low)**

| Pathways | NES | Adjust p-value | Adjust q-value |
| --- | --- | --- | --- |
| XENOBIOTIC_METABOLISM | -4.779 | 5.56E-10 | 1.29E-10 |
| BILE_ACID_METABOLISM | -4.595 | 5.56E-10 | 1.29E-10 |
| FATTY_ACID_METABOLISM | -3.396 | 5.56E-10 | 1.29E-10 |
| COAGULATION | -3.208 | 5.56E-10 | 1.29E-10 |
| PEROXISOME | -2.136 | 0.000104362 | 2.42E-05 |
| INTERFERON_GAMMA_RESPONSE | 1.377 | 0.037802419 | 0.008754244 |
| UV_RESPONSE_DN | 1.391 | 0.041283646 | 0.009560423 |
| PROTEIN_SECRETION | 1.412 | 0.036894923 | 0.008544087 |
| ANDROGEN_RESPONSE | 1.435 | 0.042050434 | 0.009737995 |
| PI3K_AKT_MTOR_SIGNALING | 1.537 | 0.014717897 | 0.003408355 |
| IL6_JAK_STAT3_SIGNALING | 1.538 | 0.018487346 | 0.00428128 |
| APICAL_JUNCTION | 1.555 | 0.001708271 | 0.0003956 |
| NOTCH_SIGNALING | 1.556 | 0.048967843 | 0.011339921 |
| INFLAMMATORY_RESPONSE | 1.567 | 0.001708271 | 0.0003956 |
| DNA_REPAIR | 1.574 | 0.004596571 | 0.001064469 |
| KRAS_SIGNALING_UP | 1.581 | 0.001708271 | 0.0003956 |
| IL2_STAT5_SIGNALING | 1.603 | 0.00125318 | 0.00029021 |
| ESTROGEN_RESPONSE_EARLY | 1.607 | 0.000937219 | 0.00021704 |
| MYOGENESIS | 1.611 | 0.001708271 | 0.0003956 |
| MYC_TARGETS_V2 | 1.618 | 0.01385899 | 0.00320945 |
| TGF_BETA_SIGNALING | 1.623 | 0.009535556 | 0.002208234 |
| APOPTOSIS | 1.640 | 0.001708271 | 0.0003956 |
| UNFOLDED_PROTEIN_RESPONSE | 1.729 | 0.000915226 | 0.000211947 |
| ALLOGRAFT_REJECTION | 1.732 | 7.51E-05 | 1.74E-05 |
| SPERMATOGENESIS | 1.742 | 0.000843193 | 0.000195266 |
| UV_RESPONSE_UP | 1.750 | 0.000140646 | 3.26E-05 |
| P53_PATHWAY | 1.773 | 2.50E-05 | 5.80E-06 |
| WNT_BETA_CATENIN_SIGNALING | 1.797 | 0.003856266 | 0.00089303 |
| HYPOXIA | 1.812 | 1.76E-05 | 4.08E-06 |
| GLYCOLYSIS | 1.887 | 1.13E-06 | 2.61E-07 |
| ESTROGEN_RESPONSE_LATE | 1.888 | 2.30E-06 | 5.33E-07 |
| ANGIOGENESIS | 2.005 | 0.000672066 | 0.000155636 |
| TNFA_SIGNALING_VIA_NFKB | 2.040 | 8.73E-09 | 2.02E-09 |
| MTORC1_SIGNALING | 2.059 | 5.32E-09 | 1.23E-09 |
| MITOTIC_SPINDLE | 2.268 | 5.56E-10 | 1.29E-10 |
| MYC_TARGETS_V1 | 2.293 | 5.56E-10 | 1.29E-10 |
| EPITHELIAL_MESENCHYMAL_TRANSITION | 2.702 | 5.56E-10 | 1.29E-10 |
| E2F_TARGETS | 3.152 | 5.56E-10 | 1.29E-10 |
| G2M_CHECKPOINT | 3.215 | 5.56E-10 | 1.29E-10 |
