# Supplementary figures and images for: A cellular senescence-related classifier based on a tumorigenesis- and immune infiltration-guided strategy can predict prognosis, immunotherapy response, and candidate drugs in hepatocellular carcinoma
Source: Front Immunol. 2022 Nov 15;13:974377. doi: 10.3389/fimmu.2022.974377 (PMC9705748; doi:10.3389/fimmu.2022.974377)

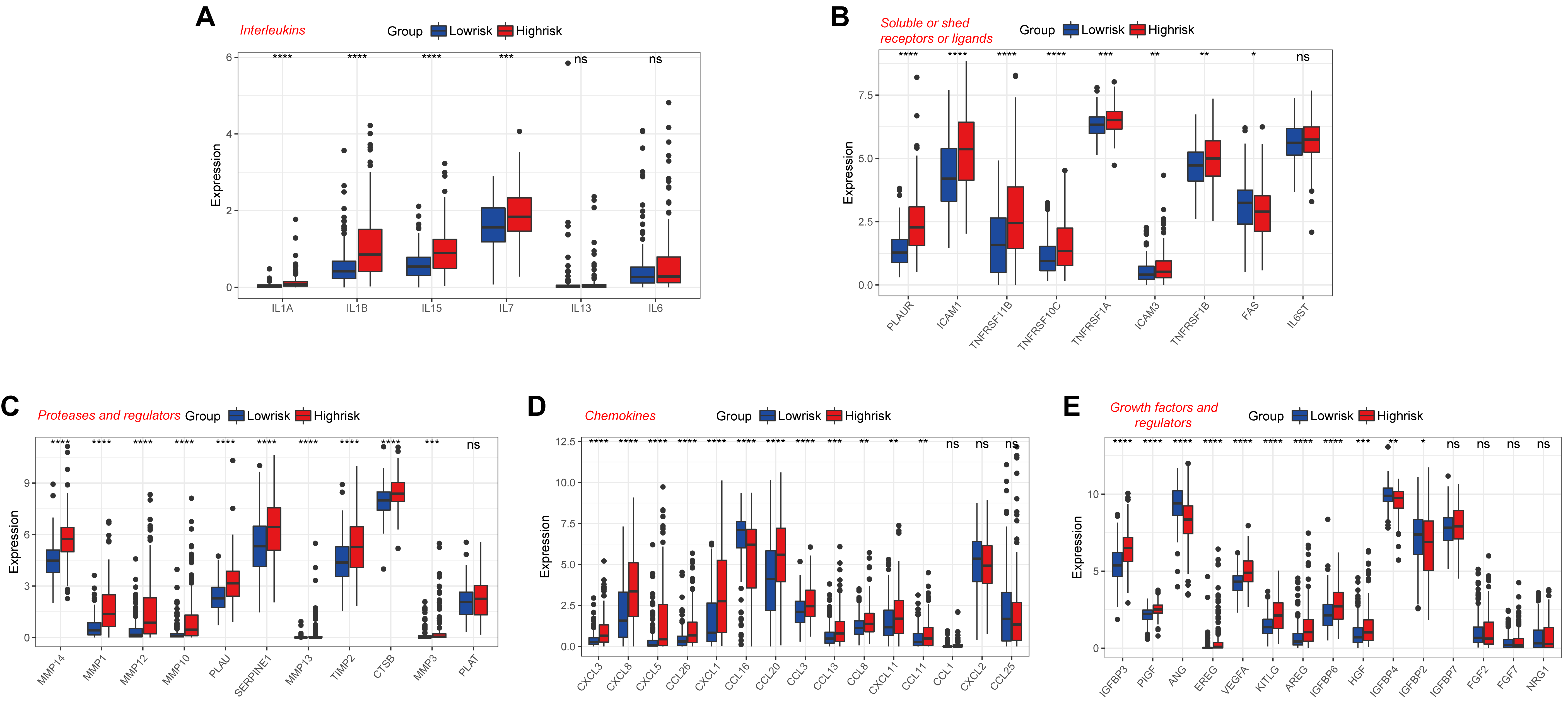

Supplement: Supplementary Figure 1 — Distinct SASP landscapes between the TIS-high and TIS-low groups. Differential expression of five types of SASP factors, including interleukins (A), soluble or shed receptors or ligands (B), proteases and regulators (C), chemokines (D), and growth factors and regulators (E), between TIS-high and TIS-low groups. [file Image_1.tif]

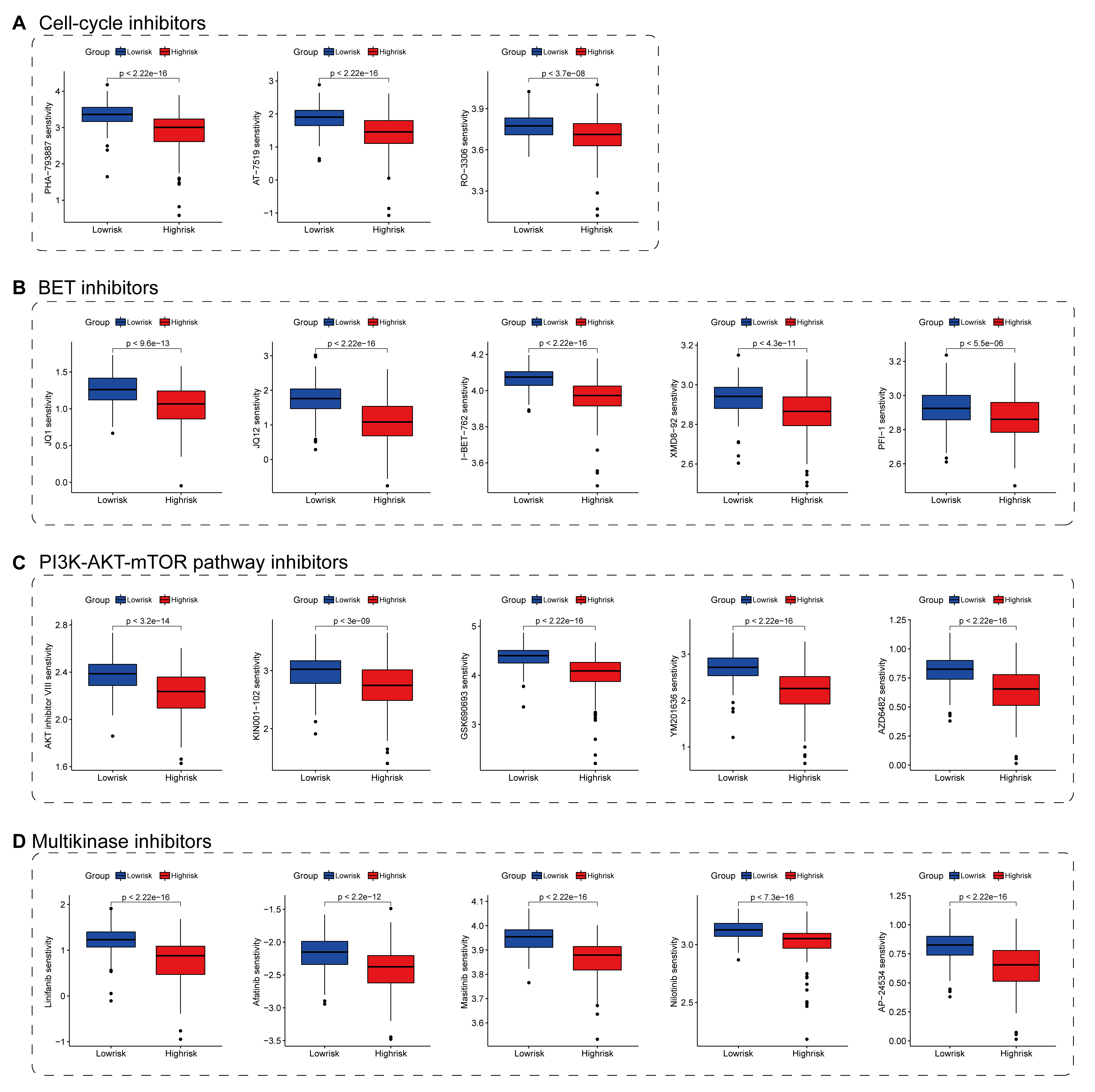

Supplement: Supplementary Figure 2 — The relationship of TIS with the drug sensitivity of four senescence-related pathway inhibitors. The distinct drug sensitivity of cell-cycle inhibitors (A), BET inhibitors (B), PI3K-AKT pathway inhibitors (C), and multikinase inhibitors (D). BET: bromodomain and extraterminal domain family. [file Image_2.tif]
